# Development of a green, concise synthesis of nicotinamide derivatives catalysed by Novozym® 435 from *Candida antarctica* in sustainable continuous-flow microreactors†

**DOI:** 10.1039/d3ra07201k

**Published:** 2024-01-02

**Authors:** Zhi-Kai Sheng, Yi Liu, Li-Hua Du, Shi-Yi Zhang, Ao-Ying Zhang, Han-Jia Xie, Hang Lin, Bing-Lin Yan, Miao-Miao Xue, Zhi-Xuan Ruan, Guo-Neng Fu, Bing-Le Pan, Tong-Yao Zhou, Xi-Ping Luo

**Affiliations:** a College of Pharmaceutical Science, ZheJiang University of Technology Zhejiang Hangzhou 310014 China orgdlh@zjut.edu.cn +86 571 88320903 +86-189-690-693-99; b Zhejiang Provincial Key Laboratory of Chemical Utilization of Forestry Biomass, Zhejiang A&F University Zhejiang Hangzhou 311300 China

## Abstract

An increasing number of studies have shown that many nicotinamide derivatives exhibited extensive biological activities, such as anti-inflammatory and antitumor activity. In this paper, a green, concise synthesis of nicotinamide derivatives in sustainable continuous-flow microreactors catalysed by Novozym® 435 from *Candida antarctica* has been developed. Application of an easily obtainable and reusable lipase in the synthesis of nicotinamide derivatives from methyl nicotinate and amines/benzylamines reacted for 35 min at 50 °C led to high product yields (81.6–88.5%). Environmentally friendly *tert*-amyl alcohol was applied as a reaction medium. Substantially shorter reaction times as well as a significant increase in the product yield were obtained as compared to the batch process. This innovative approach provides a promising green, efficient and rapid synthesis strategy for pharmaceutical synthesis and further activity research of novel nicotinamide derivatives.

## Abstract 2

A continuous production of nicotinamide derivatives was performed for the first time by exploiting the continuous-flow microreactors coupled with enzymes.

## Graphical abstract

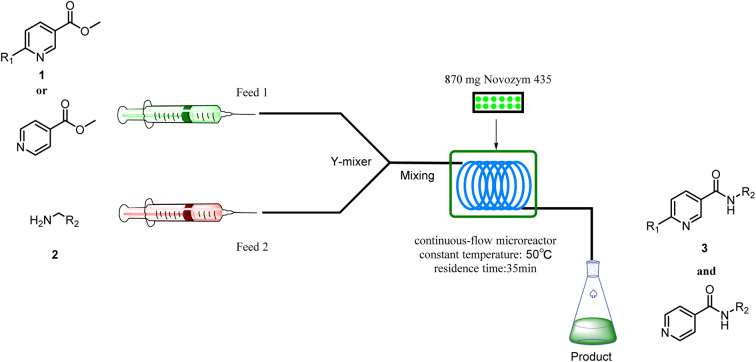

## Introduction

**Fig. 1:**
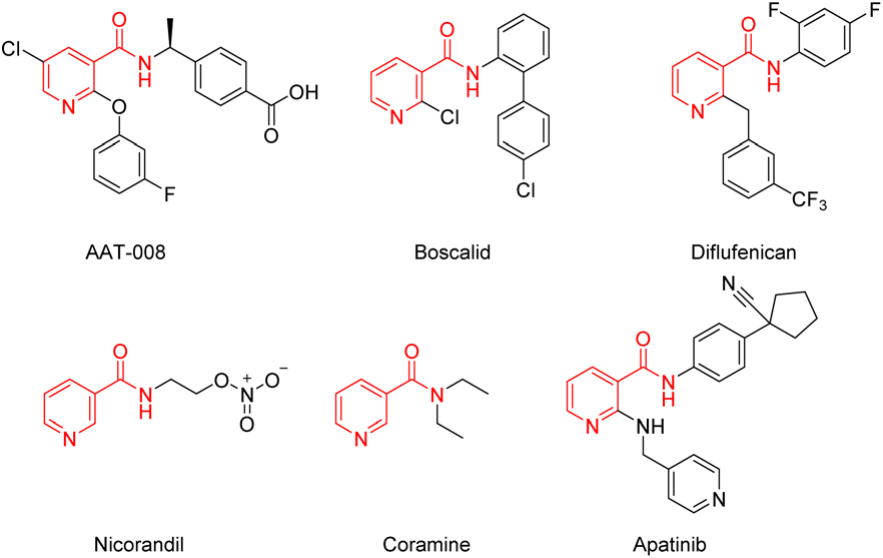
Structures of nicotinamide derivatives with potential drug activities.

Nicotinamide is a water-soluble B complex vitamin which is naturally present in animal offal, whole cereals and legumes.^1,2^ Nicotinamide derivatives have been proven to possess extensive biological activities, such as anti-inflammatory properties,^3^ vitro antimicrobial and antifungal activity,^4–7^ insecticidal^8^ and herbicidal activity.^9^ Along with an increasing focus on the utilization of natural resources in organic synthesis, nicotinamide derivatives are gaining more and more interest. Numerous nicotinamide derivatives with various substituents have been applied to medicine and agrochemistry, such as AAT-008 (EP4 receptor antagonists),^10^ boscalid and diflufenican (broad-spectrum fungicide),^11^ nicorandil (cardiovascular medicines),^12,13^ coramine (respiratory stimulant), apatinib (anti-cancer)^14,15^ (Fig. 1). These derivatives serve as crucial therapeutic agents in medicinal chemistry, highlighting the necessity for the advancement of new construction methods.

Construction of amide bonds is a vital part of the synthesis of nicotinamide derivatives. The current methods for the synthesis of amides are remarkably ordinary but at the same time widely regarded as expensive and tedious. Typical methods for amide bond formation employ stoichiometric amounts of activating reagents such as DDC (*N*,*N*′-dicyclohexylcarbodiimide), EDC (1-ethyl-3-(3′-dimethylaminopropyl)-carbodiimide hydrochloride), HATU ((1-[bis(dimethylamino)methylene]-1*H*-1,2,3-triazolo[4,5-*b*]pyridinium 3-oxide hexafluorophosphate), CDI (1,1′-carbonyldiimidazole) or BOP (benzotriazol-1-yloxy)-tris(dimethylamino)-phosphonium hexafluorophosphate), which create an activated acid that may be attacked by the nucleophilic reagent.^16–19^ The transition metal including copper (Cu),^20^ palladium (Pd),^21,22^ nickel (Ni),^23,24^ manganese (Mn),^25^ catalyzed amide bond forming reaction of esters with amines, has been developed as an advanced method in recent years. However, the use of the condensation reagents and the transition metal cause a number of problems, such as creating a considerable amount of waste products and energy, recycling and reprocessing waste complexly, and even may severe allergic reactions and cause potential safety and toxicity issues of the metal and condensation reagents.^16,26–31^ Except for precious metals, many other metal catalysts such as nickel and manganese(i) pincer complexes, usually require a series of tedious operations for preparation. Such reactions usually needed to be carried out more than 16 h at high temperatures in the presence of toluene, which is a carcinogenic and non-green solvent.^23–25^ Not surprisingly, in 2007 the American Chemical Society Green Chemistry Institute Pharmaceutical Roundtable (ACS GCIPR) voted that amide formation avoiding poor atom economy reagents as the top challenge for organic chemistry.^32,33^ Therefore, the development of a concise, green, efficient and sustainable synthetic route for the synthesis of nicotinamide derivatives is highly desirable.

Biocatalysis, using enzymes for organic synthesis, has emerged as a powerful tool for offering another potential solution for sustainable amide formation.^34,35^ Biocatalysis has outstanding advantages over chemocatalysis, including mild reaction conditions, high stereoselectivity and high catalytic efficiency.^36^ Recently, whole-cell bioconversion has been used as a novel and popular biocatalytic method to produce a range of products. For instance, *Escherichia coli* has been utilized to produce alkanes, alcohols, amides, carboxylic acids and chiral drug intermediates.^37–40^ A range of hydrolases (lipases, esterases, acylases) and the Penicillin G acylases have been widely used in industrial production of amide compounds.^18,41,42^ Enzymatic reactions are generally conducted under mild conditions (ambient temperature and barometric pressure) in aqueous solution, usually without functional-group activation, protection and deprotection steps.^43^ Although, chemo-enzymatic reaction conditions are mild, the required reaction times to obtain the desired yield at room temperature can be as long as several days. As recently shown by Zheng *et al.*, the amidase from *Pantoea* sp. (Pa-Ami) is reported for biocatalytic hydrolysis of 2-chloronicotinamide, and the product 2-chloronicoinic acid was produced as high as 370 mM with a substrate conversion of 94.2% at 40 °C in Tris–HCl buffer for 8 h.^44^ There are currently limited instances of using enzymes for the synthesis of nicotinamide derivatives. In order to explore the synthesis reactions of enzyme-catalyzed nicotinamide derivatives and improve the efficiency of traditional enzyme catalytic reactions, we focus on continuous-flow technology.

**Scheme 1:**
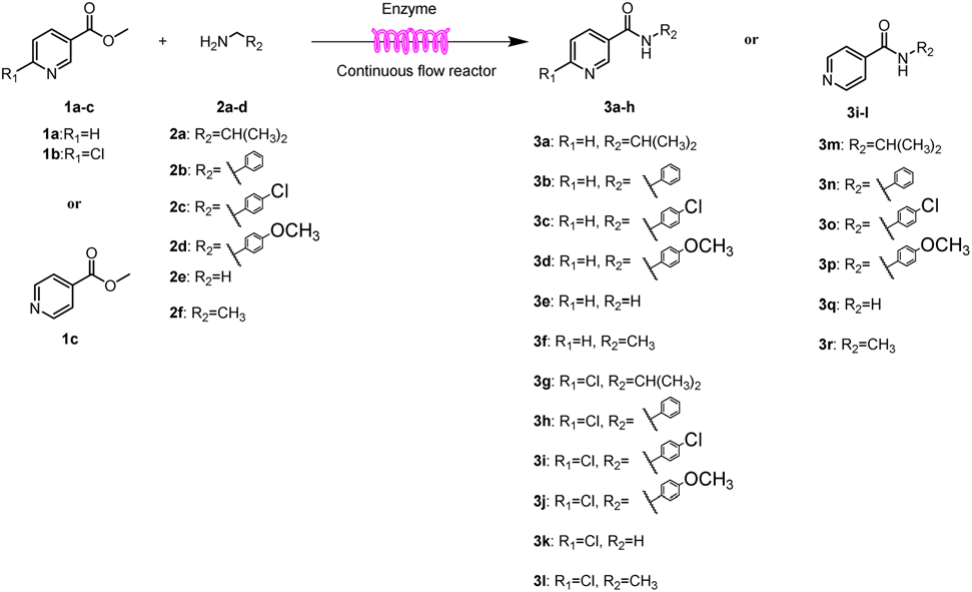
Synthesis of nicotinamide derivatives in the continuous flow microreactors.

Continuous-flow technology is showing spectacular applications in the manufacture of fine chemicals and pharmaceuticals in these few decades.^27^ In 2007, ACS GCIPR listed several key areas where research was required to facilitate the development of sustainable manufacturing, and the importance of continuous processing was acknowledged.^45^ Compared with conventional batch reactors, fundamental advantages of continuous-flow technology are precise temperature and residence time control, the enhancement of heat and mass transfer, and handling of hazardous regents in a safe way.^46–50^ In this work, we utilize immobilized enzymes in continuous-flow systems, which vastly improves reusability and significantly reduces the reaction time while reducing solvent waste and energy consumption for later solvent removal.^51,52^ The aim of this paper is to develop a green and concise method for the synthesis of nicotinamide derivatives from methyl nicotinates (methyl nicotinate, methyl 6-chloronicotinate and methyl isonicotinate) and several amines (isobutylamine, methylamine, ethylamine) and benzylamines (benzylamine, 4-chlorobenzylamine, 4-methoxybenzylamine) catalyzed by Novozym® 435 from *Candida antarctica* in continuous-flow microreactors (Scheme 1). Reaction parameters such as reaction mediums, temperature, substrate ratio, reaction flow rate/residence time were examined. The influence of reactant structure on nicotinamide derivatives synthesis reaction had also been studied. 18 nicotinamide derivatives were efficiently synthesized through this method. Compared with the same reaction we performed in the batch reactor, the reaction time was greatly shortened from 24 h to 35 min and the yield was also significantly improved.

## Results and discussion

Generally, the type of the solvent and enzyme play an important role in enzymatic reaction. The factors affecting the conversion of solvents are very manifold, including solubility of the substrate, hydrophobicity, polarity, toxicity to enzymes, and regioselectivity.^53–56^ Lipase is known to show high activity in hydrophobic solvents.^57,58^ It is necessary to choose a solvent that dissolves substrates effectively while maintaining sufficient enzyme activity. The activity and stability of enzyme can be maximized when the hydrophobicity (log *P*) of the solvent ranges from 0.6 to 3.5.^59^

Log *P* is the logarithm of the concentration ratio when water and octanol are mixed. The initial reaction rate of lipase in the hydrophobic solvent gets faster as it has a lower log *P* value and higher polarity.^57^ The ammonolysis reaction of methyl nicotinate and isobutylamine was carried out using an immobilized enzyme in the continuous flow reactors. We performed a blank control experiment without any enzyme and found that the reaction did not occur. Experiments were performed using Novozym® 435 (immobilized *Candida antarctica* lipase B) and Lipozyme TL IM (immobilized *Thermomyces lanuginosus*) in several commonly organic solvents such as *tert*-amyl alcohol, methanol, isopropanol, isobutanol, DMSO, DMF, acetonitrile, acetone, toluene. The log *P* values of the solvents used in the experiment and yields of product are shown in the Table 1. As is apparently shown in the Table 1, *tert*-amyl alcohol, isopropanol and acetonitrile show high yields in this experiment, while methanol, acetone and toluene had low yields. Among the solvents used, DMSO and DMF had almost no reaction even though had the lowest log *P*, which might be due to the inactivation of enzymes under DMSO and DMF conditions.^60^ However, *tert*-amyl alcohol is a greener solvent than acetonitrile. Therefore, *tert*-amyl alcohol is selected as the optimal reaction solvent in this experiment.

**Table 1:** The effect of reaction media and catalyst on the enzymatic synthesis of nicotinamide derivatives in continuous-flow microreactors^a^

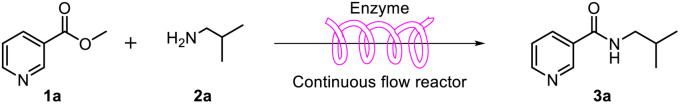				
Entry	Solvent	Catalysts	Log *P*	Yield^b^ (%)
1	*tert*-Amyl alcohol	Novozym® 435	1.04	82.4 ± 1.2
2	Methanol	Novozym® 435	−0.76	26.5 ± 0.6
3	Isopropanol	Novozym® 435	−0.39	54.3 ± 1.4
4	Isobutanol	Novozym® 435	0.69	n.d.
5	DMSO	Novozym® 435	−1.35	n.d.
6	DMF	Novozym® 435	−1.0	n.d.
7	Acetonitrile	Novozym® 435	−0.33	64.8 ± 1.5
8	Acetone	Novozym® 435	−0.16	34.5 ± 0.8
9	Toluene	Novozym® 435	2.5	18.6 ± 1.2
10	*tert*-Amyl alcohol	Lipozyme® TL IM	1.04	n.d.
11	*tert*-Amyl alcohol	None	1.04	n.d.

a General experimental conditions: in the continuous flow reactors, feed 1, 10 mL solvent contained 5.0 mmol methyl nicotinate (1a); feed 2, 10 mL solvent contained 20.0 mmol isobutylamine (2a), 50 °C, flow rate 17.8 μL min^−1^ residence time 35 min, enzyme 870 mg.

b Isolated yield. Yield: 100 × (actual received amount/ideal calculated amount). The data are presented as average ± standard deviation (SD) of triplicate experiments. n.d. means no reaction was found.

**Fig. 2:**
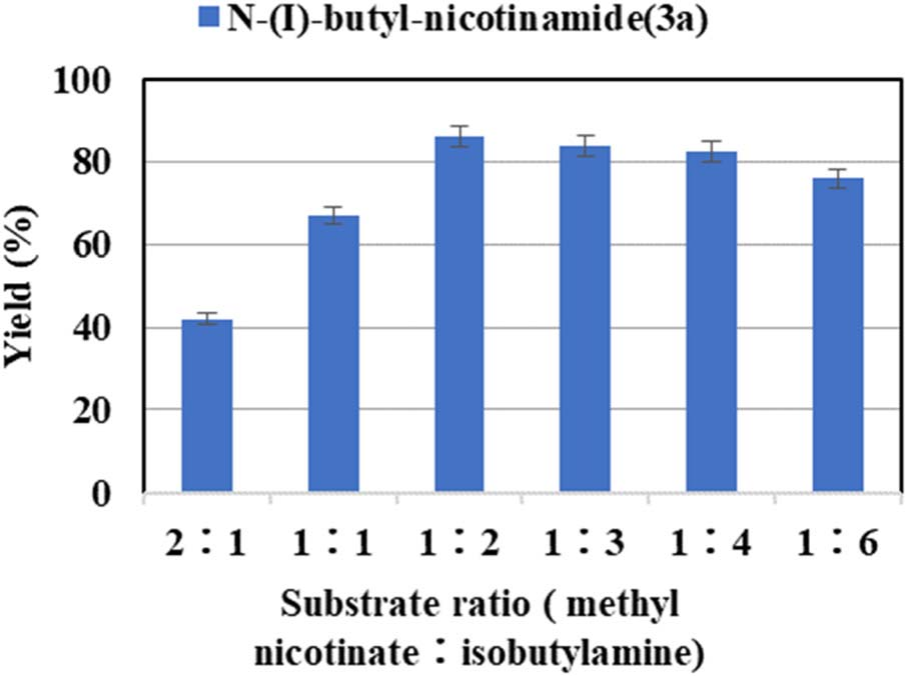
The effect of substrate ratio (methyl nicotinate : isobutylamine) on the enzymatic synthesis of nicotinamide derivatives in continuous-flow microreactors.

The theoretically perfect substrate molar ratio for reaction between methyl nicotinate and isobutylamine is 1 : 1. However, the amidation reaction is a reversible reaction. A high reaction rate can be achieved within a short period if an excessive number of reactants are used. In this study, six substrate ratios of methyl nicotinate/isobutylamine (2 : 1, 1 : 1, 1 : 2, 1 : 3, 1 : 4, 1 : 6) were investigated. As shown in Fig. 2, the yield of the target product increases gradually with the amines. The highest yield of 86.2% was obtained when the substrate molar ratio (methyl nicotinate : isobutylamine) was 1 : 2. The reason why the yield decreases while the substrate ratio was increased further is that excessive substrate concentration changed the viscosity and polarity of the solvent, interferes with mass transfer, affects the polarity of the enzyme, while reducing the economic efficiency.^53,54,57,61^ Hence, the substrate ratio of 1 : 2 was chosen the optimal ratio.

**Fig. 3:**
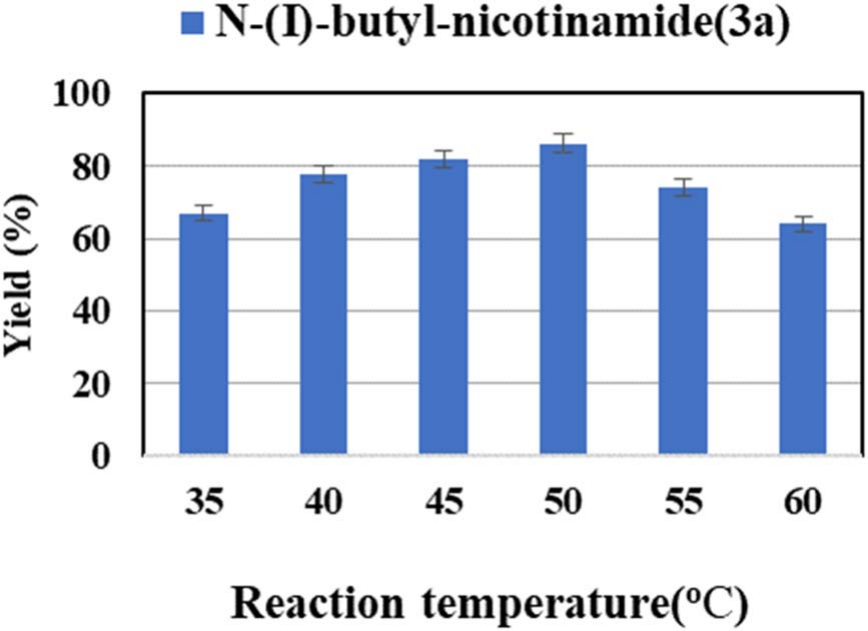
The effect of reaction temperature on the enzymatic synthesis of nicotinamide derivatives in continuous-flow microreactors.

Reaction temperature has various effects on the properties of enzymatic reactions, such as the stability and the activity of the enzyme and the viscosity of reaction media. Therefore, the reaction temperature of 35–60 °C was investigated. The flow reaction was carried out in *tert*-amyl alcohol using Novozym® 435 as catalyst. As shown in Fig. 3, the maximum yield was obtained when the reaction temperature was 50 °C. When the temperature was relatively low, the reaction rate increases with increasing temperature. However, when it exceeds 50 °C, a further increase in temperature might result in irreversible denaturation and inactivation of enzyme, thus resulting in a decrease in yield.^62^

**Fig. 4:**
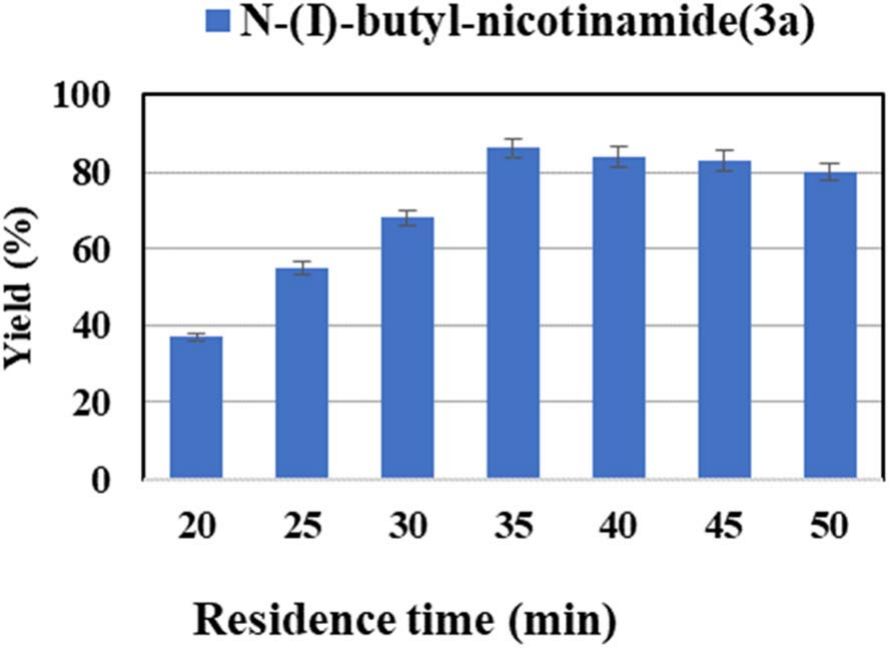
The effect of residence time on the synthesis of nicotinamide derivatives in continuous-flow microreactors.

Besides, residence time is another crucial influence parameter affecting the yield of reaction. In the continuous-flow experiments, the residence time is controlled by adjusting the flow velocity while keeping the length of the microbore tube. The residence time was investigated from 20 to 50 min (Fig. 4). The transformation and mass transfer were improved by increasing the flow velocity through the same reactor length. And a maximum yield of 86.2% was obtained at 35 min (flow rate of 17.8 μL min^−1^). This result is anticipated, as for a constant reaction temperature and velocity, a higher residence time means more time is available for the reaction to be completed.^63^ In addition, too powerful flow would denature the enzyme.^64^ Therefore, 35 min was selected as the optimal residence time for the following studies.

**Fig. 5:**
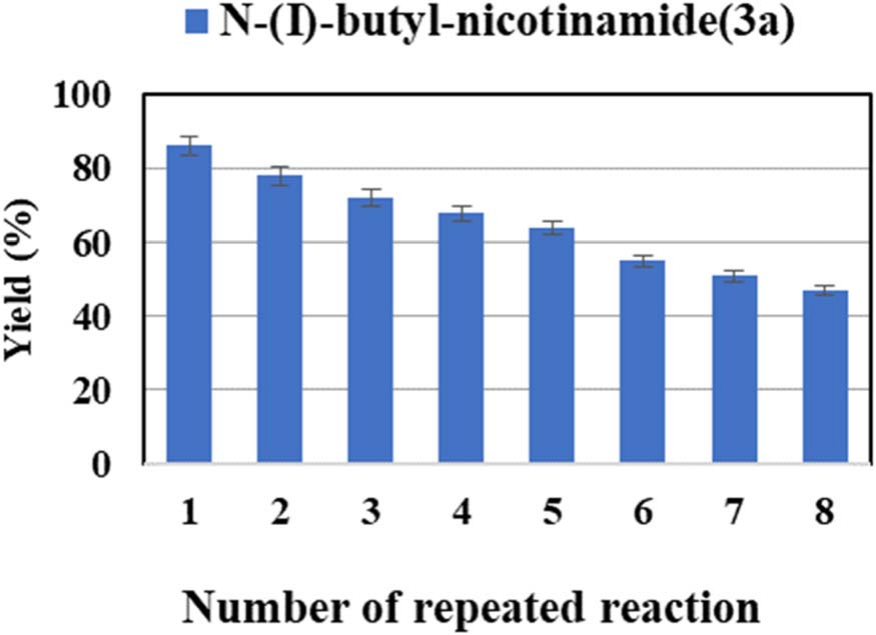
The effect of enzyme reusability on the enzymatic synthesis of nicotinamide derivatives in continuous-flow microreactors.

It is very important for a reaction system with application potentials that the biocatalyst exhibits good stability and reusability. What's more, the cost can be reduced by recycling and reusing the immobilized lipase. We tested the reusability of Novozym® 435 in the continuous-flow reactor. The enzyme was reused eight times, it was found that the catalytic yield of the last catalysis could still exceed 45% (Fig. 5). These results show a satisfactory reusability of the enzyme due to its stability. Such decreased activity after successive reaction cycles could be due to the desorption of immobilized lipase during the continuous flow. In addition, the accumulation of residual starting material and/or products remaining in the biocatalyst microenvironment might also lead to enzyme inactivation or desorption from the support surface.^65–67^

Then, to explore the influence of enzymatic reactions in continuous-flow systems and batch systems, the enzymatic reactions in different reactors were carried out. Space-time yield (STY), a common parameter, is frequently used to evaluate the productivity of different reaction system normalized to 1 liter volume (g h^−1^ L^−1^).^68^ Since batch and continuous-flow system setups have a completely different geometry, a direct comparison based on conversion or yield is simply not possible.^68^ On the contrary, the calculation of the STY is able to compare between the different systems equitably. Besides, the STY is a reasonable and important quantity for the comparability of continuously operated flow processes.^63,69,70^
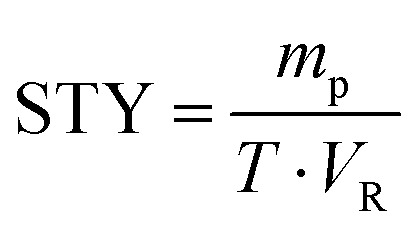
where *m*_p_ is the mass of the generated product (g), *T* is the residence time (h), and *V*_R_ is the reactor volume (L).

As shown in Table 2, the product yield obtained by the continuous-flow micromixer is obviously better than that by the traditional shaker reactor. In the traditional batch bioreactor (Method B), the enzymatic reaction took about 24 hours to reach the expected yield while the reaction in the continuous-flow microreactor (Method A) obtained better yields in 35 min.

**Table 2:** Enzymatic synthesis of nicotinamide derivatives in the continuous-flow microreactor or the batch reactor^a^

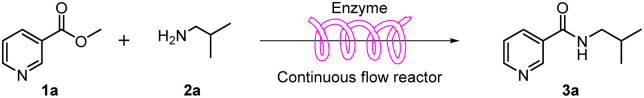			
Entry	Method	STY (g L^−1^ h^−1^)	Yield^b^ (%)
1	A	26.13	86.2 ± 1.5
2	B	0.50	67.1 ± 0.8

a General experimental conditions: Method A: continuous flow reactors, feed 1, dissolve 5 mmol of methyl nicotinate (1a) in 10 mL *tert*-amyl alcohol; feed 2, dissolve 10 mmol of isobutylamine (2a) in 10 mL *tert*-amyl alcohol, flow rate 17.8 μL min^−1^, residence time 35 min, enzyme 870 mg, 50 °C. Method B: shaker reactors, add 5 mmol of methyl nicotinate (1a), 10 mmol of isobutylamine (2a) and 20 mL *tert*-amyl alcohol to a 50 mL Erlenmeyer flask, enzyme 870 mg, 160 rpm, 50 °C, 24 h.

b Isolated yield. Yield: 100 × (actually obtained amount/calculated amount). The data are presented as average ± SD of triplicate experiments.

After optimizing the reaction conditions, in order to explore the scope and limitations of the enzymatic ammonolysis reaction, the reactions of methyl nicotinate derivatives (methyl nicotinate (1a), methyl 6-chloronicotinate (1b) and methyl isonicotinate (1c)) with amines (isobutylamine, methylamine, ethylamine) and benzylamines (benzylamine, 4-chlorobenzylamine, 4-methoxybenzylamine) was carried out in continuous-flow microreactors and batch reactors. As shown in Table 3, the corresponding compounds were synthesized parallelly in a single experiment, which proves that the enzymatic reaction has good scalability. We found that the yield of ammonolysis reaction with aliphatic amine (*e.g.*, entry 1, 86.2%) was significantly higher than which with benzylamine (*e.g.*, entry 2, 68.5%). Meanwhile, benzylamine with an electron-donating group (*e.g.*, entry 4, 71.2%) were more favourable for the reaction than benzylamine with an electron-withdrawing group (*e.g.*, entry 3, 65.2%), which conform to the reaction mechanism of nucleophilic substitution.

**Table 3:** The effect of substrate structure to the enzymatic synthesis of coumarin carboxamide derivatives under continuous-flow conditions^a^

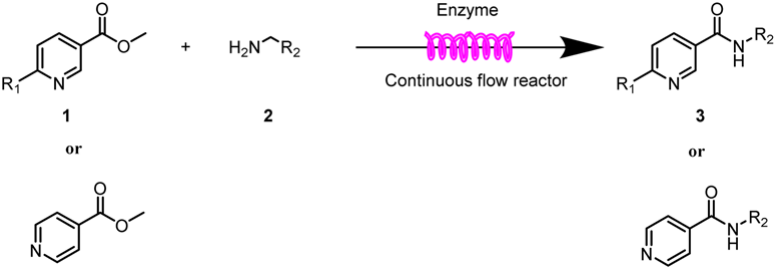						
Entry	Methyl nicotinate	*R*_2_	Method	Time	Product	Yield^b^ (%)
1	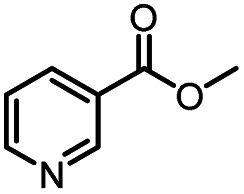	CH(CH_3_)_2_	A	35 min	3a	86.2 ± 1.5
			B	24 h		67.1 ± 0.8
2	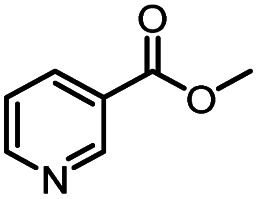	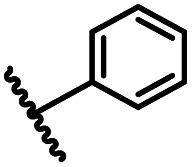	A	35 min	3b	68.5 ± 0.8
			B	24 h		54.2 ± 0.9
3	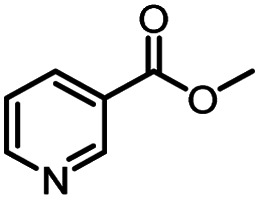	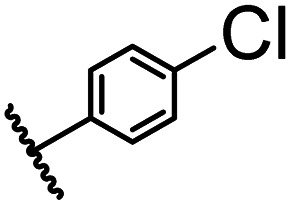	A	35 min	3c	65.2 ± 1.5
			B	24 h		54.3 ± 1.2
4	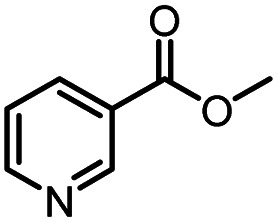	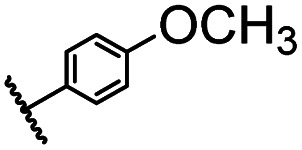	A	35 min	3d	71.2 ± 0.8
			B	24 h		62.8 ± 0.9
5	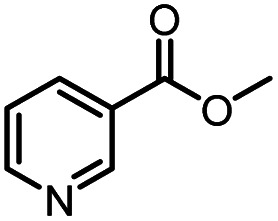	H	A	35 min	3e	86.8 ± 0.4
			B	24 h		68.7 ± 0.9
6	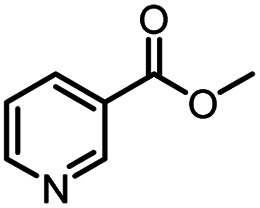	CH_3_	A	35 min	3f	83.3 ± 0.9
			B	24 h		75.9 ± 1.2
7	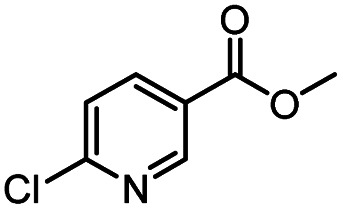	CH(CH_3_)_2_	A	35 min	3g	88.5 ± 0.7
			B	24 h		77.4 ± 1.5
8	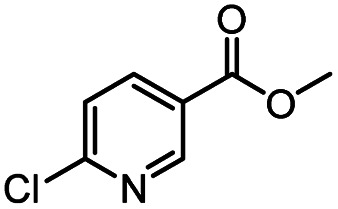	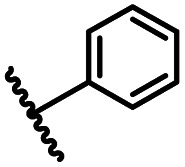	A	35 min	3h	69.4 ± 1.8
			B	24 h		58.5 ± 0.7
9	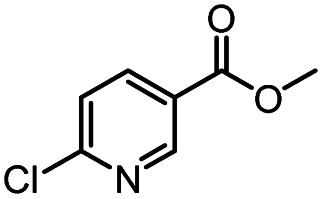	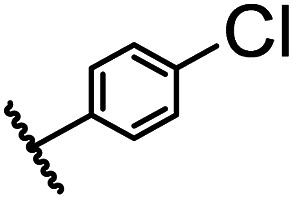	A	35 min	3i	65.4 ± 0.5
			B	24 h		55.9 ± 0.8
10	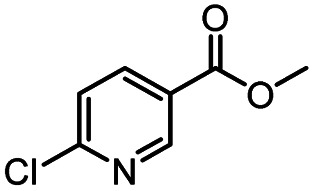	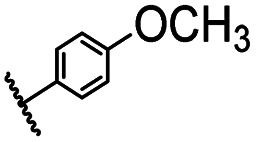	A	35 min	3j	72.5 ± 0.7
			B	24 h		61.5 ± 0.5
11	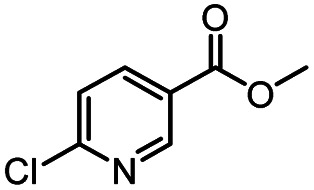	H	A	35 min	3k	81.6 ± 0.7
			B	24 h		67.5 ± 0.7
12	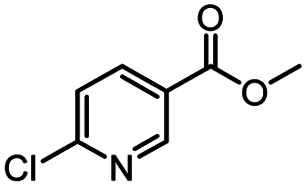	CH_3_	A	35 min	3l	85.6 ± 1.5
			B	24 h		65.9 ± 0.7
13	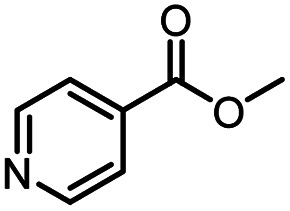	CH(CH_3_)_2_	A	35 min	3m	85.1 ± 1.2
			B	24 h		69.8 ± 0.7
14	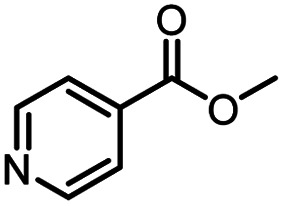	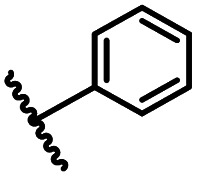	A	35 min	3n	75.5 ± 0.7
			B	24 h		57.8 ± 0.5
15	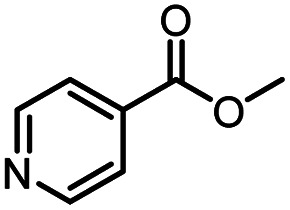	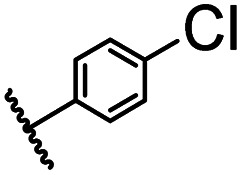	A	35 min	3o	73.8 ± 0.9
			B	24 h		52.5 ± 0.6
16	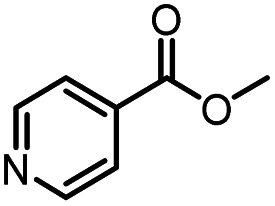	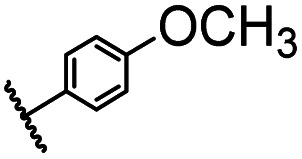	A	35 min	3p	75.4 ± 1.5
			B	24 h		58.6 ± 0.8
17	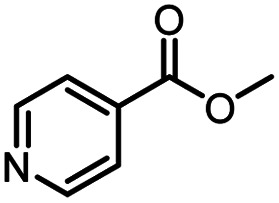	H	A	35 min	3q	81.6 ± 1.2
			B	24 h		66.5 ± 0.7
18	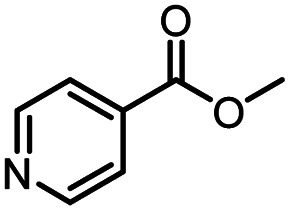	CH_3_	A	35 min	3r	82.2 ± 0.7
			B	24 h		62.3 ± 0.6

a General experimental conditions: Method A: continuous flow reactors, feed 1, dissolve 5 mmol of methyl nicotinate derivatives in 10 mL *tert*-amyl alcohol; feed 2, dissolve 10 mmol of amines in 10 mL *tert*-amyl alcohol, flow rate 17.8 μL min^−1^, residence time 35 min, enzyme 870 mg, 50 °C. Method B: shaker reactors, add 5 mmol of methyl nicotinate derivatives, 10 mmol of amines and 20 mL *tert*-amyl alcohol to a 50 mL Erlenmeyer flask, enzyme 870 mg, 160 rpm, 50 °C, 24 h.

b Isolated yield. Yield: 100 × (actually obtained amount/calculated amount). The data are presented as average ± SD of triplicate experiments.

To demonstrate the greener aspect of the current synthetic method, green chemistry metrics of the compound (3a) were calculated and evaluated. Several green metrics, including atom economy (AE), carbon efficiency (CE), atom efficiency, reaction mass efficiency (RME), overall efficiency (OE), process mass intensity (PMI) and E-factor have been evaluated for the model compound (3a), which empowers us to assess the present synthetic method in terms of waste, carbon efficiency and energy usage. The calculated values of the green metrics of all the synthesized compounds are summarized in Table S1.†. Evaluation showed an E-factor of 23.39 kg waste/1 kg product, reaction mass efficiency (54.20%), atom economy (84.76%), atom efficiency (73.06%), process mass intensity (24.49), overall efficiency (63.95%) and 100% carbon efficiency for the synthesis of 3a. The calculated values endorse this presented method as green and acceptable.

## Experimental

**Fig. 6:**
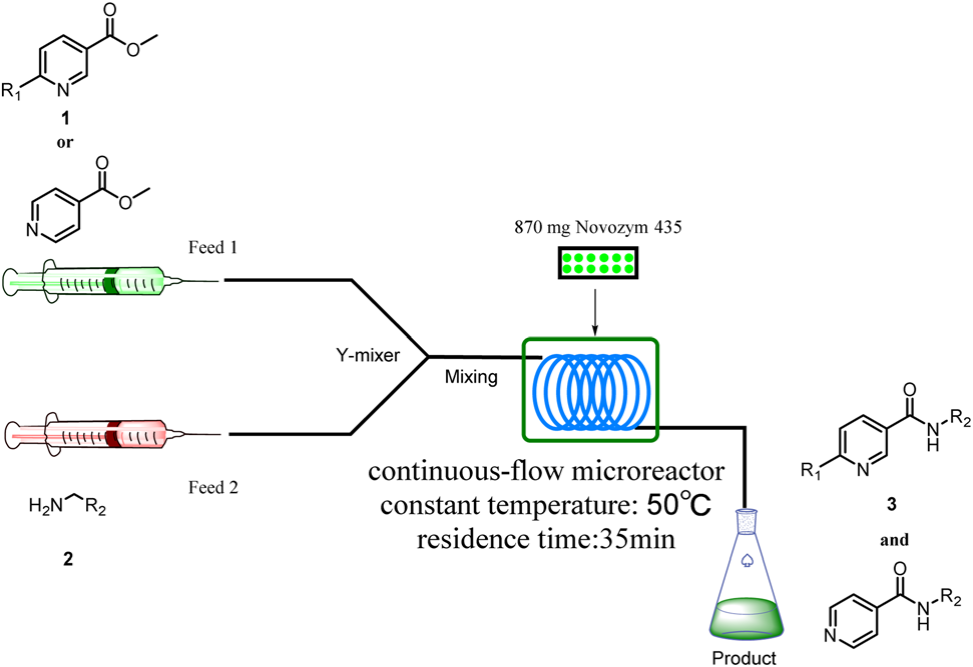
The equipment diagram for the synthesis of nicotinamide derivatives in the continuous-flow microreactor catalyzed by lipase Novozym® 435.

The equipment diagram for the synthesis of nicotinamide derivatives in the continuous-flow microreactor is depicted in Fig. 6. The experimental setup is made of a syringe pump (Harvard Apparatus Dr 2000), two substrate injectors, a Y-mixer, a flow reactor with 100 cm × 2 mm PFA tubing and a product collector. The silica gel tubing was filled with 870 mg Novozym® 435 (the lower limit of reactivity was 9000 PLU g^−1^) and placed in a thermostat water bath to maintain temperature at 50 °C. A total of 5 mmol of methyl nicotinate derivatives were dissolved in 10 mL *tert*-amyl alcohol (feed 1), and 10 mmol of amines were dissolved in 10 mL *tert*-amyl alcohol (feed 2). Feed 1 and 2 were delivered to the Y-mixer at a flow rate of 17.8  μL min^−1^ with a residence time of 35 min. The resulting stream was connected to a sample vial to collect the final mixture. The main products were separated by silica gel chromatography and were confirmed by ^1^H NMR, ^13^C NMR.

## Conclusions

Nicotinamide derivatives have been used as antitumor drugs and cardiovascular medicines. Herein, a continuous flow platform for the synthesis of valuable nicotinamide derivatives has been developed. 18 nicotinamide derivatives from methyl nicotinates (methyl nicotinate, methyl 6-chloronicotinate and methyl isonicotinate) and several amines (isobutylamine, methylamine, ethylamine) and benzylamines (benzylamine, 4-chlorobenzylamine and 4-methoxybenzylamine) were synthetized catalyzed by Novozym® 435 in continuous-flow microreactors. The reaction parameters such as reaction medium, effect of temperature, residence time, molar ratio of substrates, and enzyme reusability were systematically studied. Compared to reported works, the remarkable features of this research include shorter reaction time (35 min), mild and green reaction conditions (*tert*-amyl alcohol) and reusable and recyclable catalyst. The study of our work provides an important strategy for the concise and efficient synthesis of nicotinamide derivatives, which build a library of related compounds for further research on novel nicotinamide pharmaceuticals and drug intermediates screening.

## Author contributions

Z.-K. S., Y. L. and L.-H. D.: subject selection, experimental design, drafted and revised the manuscript; Z.-K. S, Y. L.: background research and experimental optimization; S.-Y. Z., A.-Y. Z., H.-J. X. and H. L.: collecting data; B.-L. Y., M.-M. X., Z.-X. R., G-N. F., B.-L. P., T.-Y. Z. and X.-P. L.: analyze the data and revise the manuscript. All authors read and approved the final manuscript.

## Conflicts of interest

There are no conflicts to declare.

## Supplementary Material

RA-014-D3RA07201K-s001
