# Supplementary material for: Development of a green, concise synthesis of nicotinamide derivatives catalysed by Novozym® 435 from Candida antarctica in sustainable continuous-flow microreactors
Source: RSC Adv. 2024 Jan 2;14(1):131–8. doi: 10.1039/d3ra07201k (PMC10758761; doi:10.1039/d3ra07201k)
Supplement: RA-014-D3RA07201K-s001 [file RA-014-D3RA07201K-s001.pdf]

Electronic Supplementary Material (ESI) for RSC Advances.
This journal is © The Royal Society of Chemistry 2023

Supporting Information for

Development of a green, concise Synthesis of Nicotinamide Derivatives Catalysed by Novozym® 435 from *Candida antarctica* in Sustainable Continuous-Flow Microreactors

Yi Liu, [#a] Zhi-Kai Sheng, [#a] Li-Hua Du, [*a] Shi-Yi Zhang, [a] Ao-Ying Zhang, [a] Han-Jia Xie, [a] Hang Lin, [a] Bing-Lin Yan, [a] Miao-Miao Xue, [a] Zhi-Xuan Ruan, [a] Guo-Neng Fu, [a] Bing-Le Pan, [a] Tong-Yao Zhou, [a] and Xi-Ping Luo*[b]

[a] College of Pharmaceutical Science, ZheJiang University of Technology, Hangzhou, 310014, China.

[b] Zhejiang Provincial Key Laboratory of Chemical Utilization of Forestry Biomass, Zhejiang A&F University, Hangzhou, 311300, China.

*Corresponding author. Fax: +86 18969069399. E-mail: orgdlh@zjut.edu.cn; orgdlh@gmail.com; luoxiping@zafu.edu.cn

[#] These authors contributed equally to this work and should be considered co-first authors.

Materials

All chemicals in this study were obtained from commercial sources and did not require further purification. Lipozyme TL IM (immobilized *Thermomyces lanuginosus*), Novozym® 435 (immobilized *Candida antarctica* lipase B) were purchased from Novo Nordisk Bioindustry (Copenhagen, Denmark). Methyl nicotinate, methyl 6-chloronicotinate, isobutylamine, 4-methoxybenzylamine, methylamine solution, ethylamine were purchased from Aladdin (Shanghai, China). Benzylamine, 4-chlorobenzylamine were purchased from Energy Chemical (Shanghai, China). Methyl isonicotinate was purchased from Macklin (Shanghai, China). Harvard Instrument PHD 2000 syringe pump was purchased from Harvard University (Holliston, Massachusetts, USA). The flow reactor and Y-mixer were purchased from Beijing Haigui Medical Engineering Design Co., Ltd (Beijing China).

Purification of the product

When the conversion of the nicotinamide derivative reaches a maximum (determined by TLC), the reaction is terminated by filtering the enzyme, and the tert-amyl alcohol solvent is rotary evaporated under reduced pressure. The product is separated by silica gel chromatography (mobile phase petroleum ether / ethyl acetate, 5/1 to 1/1). Purification was monitored by TLC. The graded fractions containing the major product were combined, the solvent evaporated and the residue analyzed by 1H NMR, 13C NMR.

Experimental setup

A continuous-flow microreactor equipment diagram which was used for synthesis of nicotinamide derivatives from methyl nicotinate derivatives and amines catalyzed by Novozym® 435 is described in Figure S1. The experimental setup consists of a syringe pump, two substrate injectors, Y-shaped mixers (φ= 1.8 mm) and a product collector. Syringe pumps (Harvard apparatus PHD 2000) were used to introduce separate feed streams to the flow reactor with 100cm × 2 mm PFA tubing. Silica gel tubes were filled with Novozym® 435 and immersed in a constant temperature water bath to control the temperature. A total of 5 mmol of methyl nicotinate derivatives were dissolved in 10 mL of tert-amyl alcohol (feed 1), and 10 mmol of amines were dissolved in 10 mL of tert-amyl alcohol (feed 2). Feeds 1 and 2 were placed in separate 10 mL feeders and mixed at a flow rate of 17.8 μL min-1 in a Y-mixer at 50 °C. The resulting stream (17.8 μL min-1) was connected to a sample vial for collection of the final mixture.

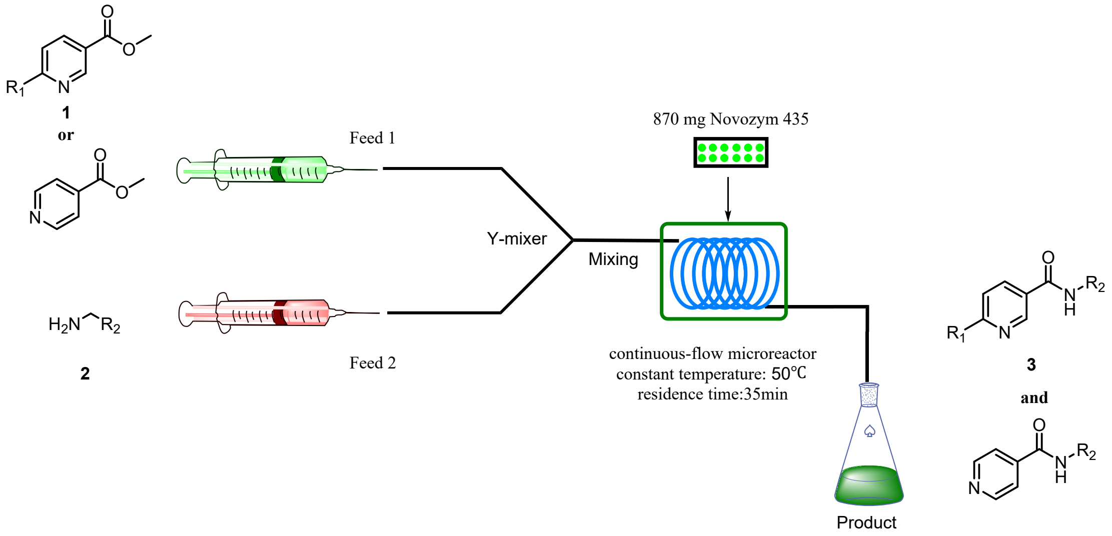

Figure S1. The equipment diagram for the synthesis of nicotinamide derivatives in the continuous-flow microreactor catalyzed by lipase Novozym® 435.

General Procedure for the synthesis of nicotinamide derivatives from methyl nicotinate derivatives and amines catalyzed by Novozym® 435 in Continuous-Flow Microreactors

Method A: 5.0 mmol of the methyl nicotinate derivatives was dissolved in 10 mL *tert*-amyl alcohol (feed A, ~0.5 M) and 10.0 mmol amine were dissolved in 10 mL *tert*-amyl alcohol (feed B; ~1.0 M). Novozym® 435 (0.87 g) were filled in PFA reactor coil (inner diameter ID= 2.0 mm, length = 100cm.). Streams A and B were mixed together at a flow rate of 8.9 μL min^{-1} in a Y-mixer at 50 °C and the resulting stream (17.8 μL min^{-1}) was connected to a sample vial which was used to collect the final mixture. The final mixture was then evaporated, and the residue was submitted to column chromatography on silica gel (200-300 mesh). The crude product was purified by silica gel column chromatography with a petroleum ether / ethyl acetate gradient from 5:1 to 1:1. The purification was monitored by TLC. The fractions containing the main products were pooled, the solvent evaporated, and the residue analyzed by ^{1}H NMR, and ^{13}C NMR.

General Procedure for the synthesis of nicotinamide derivatives from methyl nicotinate derivatives and amines catalyzed by Novozym® 435 under Shaker Conditions

Method B: methyl nicotinate derivatives (5.0 mmol) and amine (10.0 mmol) were added to 20 mL *tert*-amyl alcohol. The biocatalyst Novozym® 435 (0.87 g) was then added and the suspension maintained at 50 °C for 24h under Shaker Conditions (200 r·min^{-1}). The mixture was cooled and filtered. Then evaporated under reduced pressure and the residue was submitted to column chromatography on silica gel (200– 300 mesh). The crude product was purified by silica gel column chromatography with a petroleum ether /ethyl acetate gradient from 5:1 to 1:1. The purification was monitored by TLC. The fractions containing the main products were pooled, the solvent evaporated, and the residue analyzed by ^{1}H NMR, and ^{13}C NMR.

Calculation of Green Chemistry Metrics. [1-3]

The following formulae were used for evaluating green chemistry metrics such as atom economy (AE), carbon efficiency (CE), atom efficiency, reaction mass efficiency (RME), overall efficiency (OE), process mass intensity (PMI) and E-factor.

1. % Atom Economy (AE) *= (Mol Wt. of desired product/ Mol Wt. of all reactants) × 100*

2. % Carbon Efficiency (CE) *= (Amount of carbon in the product)/ (Total no. of carbon present in reactant) × 100*

3. % Atom efficiency *= (Yield of the product) /Atom economy) × 100*

4. Reaction Mass Efficiency (%) = (RME) *= (Mass of isolated product)/ Total mass of reactants) × 100*

5. Overall Efficiency (%) = (OE) *= (RME/AE) × 100*

6. Process Mass Intensity (%) = (PMI) *= (Total mass of input materials in a process)/ Mass of product) × 100*

7. ***E-Factor*** *= PMI-1 = (Mass of waste) / Mass of product)*

Table S1. Evaluation of green chemistry metrics for the synthesized compound **3a**

Reactant 1	Methyl nicotinate	0.686g	5 mmol	FW 137.14
Reactant 2	Isobutylamine	0.731g	10 mmol	FW 73.14
Solvent	*Tert*-amyl alcohol	16.520g (20 ml)	------	
Enzyme	Novozym® 435	0.870g	------	
Product	N-isobutyl-nicotinamide	0.768g	4.31 mmol	FW 178.24

Product yield = 86.2%
E-factor= (0.686+ 0.731+0.870+16.52 - 0.768) /0.768 x100%= 23.49 Kg waste/1Kg product.
Atom economy = (178.24/210.28) x100% = 84.76%
Atom efficiency = 86.2% x 84.76% /100% = 73.06%
Carbon efficiency = 10/10 x100% = 100%
Reaction mass efficiency = 0.768 / (0.686+ 0.731) g x 100% =54.20%
Overall Efficiency = 54.20% /84.76% x100% = 63.95%
Process Mass Intensity = (0.686+ 0.731+0.870+16.52) /0.768 = 24.49 kg /kg of the product.

Experimental data of products

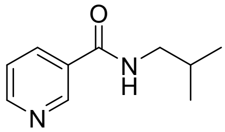

N-isobutyl-nicotinamide (3a). White solid. ^{1}H NMR (400 MHz, DMSO-d_6) δ 9.00 (d, J = 2.2 Hz, 1H), 8.73 – 8.63 (m, 2H), 8.18 (dt, J = 7.9, 2.0 Hz, 1H), 7.50 (dd, J = 7.9, 4.8 Hz, 1H), 3.10 (dd, J = 7.0, 5.8 Hz, 2H), 1.85 (hept, J = 6.7 Hz, 1H), 0.90 (d, J = 6.7 Hz, 6H). ^{13}C NMR (101 MHz, DMSO-d_6) δ 165.30, 152.16, 148.79, 135.40, 130.65, 123.89, 47.16, 28.54, 20.67.

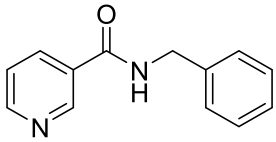

N-benzyl-nicotinamide(3b). White solid. ^{1}H NMR (400 MHz, DMSO-d6) δ 9.30 (t, J = 6.0 Hz, 1H), 9.12 (d, J = 2.3 Hz, 1H), 8.73 (dd, J = 4.8, 1.7 Hz, 1H), 8.27 (dt, J = 7.9, 2.0 Hz, 1H), 7.51 (dd, J = 8.0, 4.8 Hz, 1H), 7.40 – 7.30 (m, 4H), 7.30 – 7.21(m, 1H), 4.55 (d, J = 6.0 Hz, 2H). ^{13}C NMR (101 MHz, DMSO-d6) δ 165.35, 152.36, 148.95, 139.76, 135.50, 130.30, 128.80, 127.76, 127.31, 123.92, 43.17.

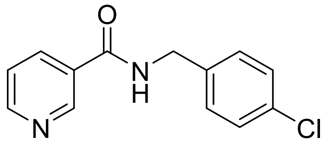

N-[(4-chlorophenyl) methyl] pyridine-3-carboxamide(3c). White solid. ^{1}H NMR (400 MHz, DMSO-d_6) δ 9.28 (t, J = 5.9 Hz, 1H), 9.05 (dd, J = 2.4, 0.9 Hz, 1H), 8.72 (dd, J = 4.8, 1.7 Hz, 1H), 8.23 (ddd, J = 7.9, 2.3, 1.7 Hz, 1H), 7.53 (ddd, J = 8.0, 4.8, 0.9 Hz, 1H), 7.44 – 7.33 (m, 4H), 4.49 (d, J = 5.9 Hz, 2H). ^{13}C NMR (101 MHz, DMSO-d_6) δ 164.66, 151.79, 148.22, 138.15, 134.81, 131.19, 129.45, 128.96, 128.08, 123.30, 41.80.

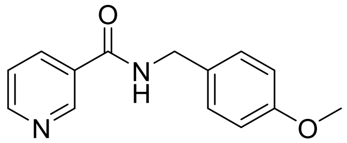

N-[(4-methoxyphenyl) methyl] pyridine-3-carboxamide(3d). White solid. ^{1}H NMR (400 MHz, DMSO-d_6) δ 9.18 (t, J = 5.9 Hz, 1H), 9.04 (dd, J = 2.4, 0.9 Hz, 1H), 8.71 (dd, J = 4.8, 1.7 Hz, 1H), 8.22 (dt, J = 8.0, 1.9 Hz, 1H), 7.51 (ddd, J = 8.0, 4.8, 0.9 Hz, 1H), 7.31 – 7.23 (m, 2H), 6.94 – 6.86 (m, 2H), 4.44 (d, J = 5.9 Hz, 2H), 3.73 (s, 3H). ^{13}C NMR (101 MHz, DMSO-d_6) δ 165.12, 158.73, 152.34, 148.89, 135.45, 131.71, 130.32, 129.14, 123.93, 114.19, 55.52, 42.57.

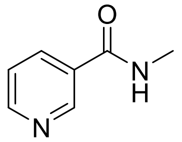

N-methyl nicotinamide(3e). White power. ^{1}H NMR (400 MHz, DMSO-d_6) δ 8.99 (dd, J = 2.3, 0.9 Hz, 1H), 8.70 (dd, J = 4.8, 1.7 Hz, 1H), 8.64 (s, 1H), 8.17 (dt, J = 7.9, 2.0 Hz, 1H), 7.50 (ddd, J = 7.9, 4.8, 1.0 Hz, 1H), 2.81 (d, J = 4.6 Hz, 3H). ^{13}C NMR (101 MHz, DMSO-d_6) δ 165.62, 152.19, 148.72, 135.25, 130.40, 123.92, 26.65.

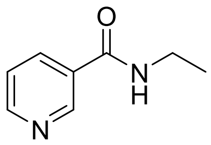

N-ethyl nicotinamide(3f). White power. ^{1}H NMR (400 MHz, DMSO-d_6) δ 9.04 – 8.98 (m, 1H), 8.68 (dt, J = 4.8, 2.4 Hz, 2H), 8.18 (dt, J = 7.9, 2.0 Hz, 1H), 7.49 (ddd, J = 7.9, 4.8, 0.9 Hz, 1H), 3.31 (qd, J = 7.2, 5.5 Hz, 2H), 1.13 (t, J = 7.3 Hz, 3H). ^{13}C NMR (101 MHz, DMSO-d_6) δ 164.96, 152.13, 148.77, 135.30, 130.53, 123.84, 34.55, 15.09.

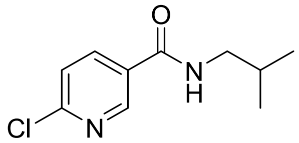

6-chloro-N-isobutylnicotinamide(3g). White solid.^{1}H NMR (400 MHz, DMSO-d_6) δ 8.83 (dd, J = 2.4, 0.7 Hz, 1H), 8.72 (t, J = 5.9 Hz, 1H), 8.24 (dd, J = 8.3, 2.5 Hz, 1H), 7.64 (dd, J = 8.3, 0.7 Hz, 1H), 3.10 (dd, J = 7.0, 5.8 Hz, 2H), 1.84 (dq, J = 13.5, 6.7 Hz, 1H), 0.90 (d, J = 6.7 Hz, 6H). ^{13}C NMR (101 MHz, DMSO-d_6) δ 164.17, 152.83, 149.37, 139.04, 130.04, 124.53, 47.23, 28.50, 20.66.

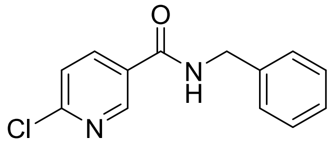

N-benzyl-6-chloro-nicotinamide(3h). White solid. ^{1}H NMR (400 MHz, DMSO-d_6) δ 9.31 (t, J = 6.0 Hz, 1H), 8.89 (d, J = 2.5 Hz, 1H), 8.29 (dd, J = 8.3, 2.5 Hz, 1H), 7.67 (d, J = 8.3 Hz, 1H), 7.34 (d, J = 4.4 Hz, 4H), 7.31 – 7.22 (m, 1H), 4.51 (d, J = 5.9 Hz, 2H). ^{13}C NMR (101 MHz, DMSO-d_6) δ 164.18, 153.06, 149.49, 139.53, 139.12, 129.68, 128.83, 127.80, 127.39, 124.63, 43.18.

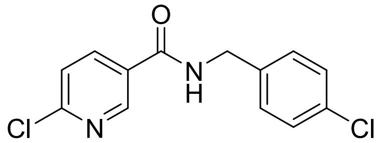

6-Chloro-N-(4-chloro-benzyl)-nicotinamide(3i). White solid. ^{1}H NMR (400 MHz, DMSO-d_6) δ 9.33 (t, J = 5.9 Hz, 1H), 8.89 (d, J = 2.5 Hz, 1H), 8.28 (dd, J = 8.3, 2.5 Hz, 1H), 7.66 (dd, J = 8.3, 1.9 Hz, 1H), 7.43 – 7.33 (m, 4H), 4.49 (d, J = 5.9 Hz, 2H). ^{13}C NMR (101 MHz, DMSO-d_6) δ 164.25, 153.14, 149.49, 139.10, 138.59, 131.95, 129.69, 129.54, 128.75, 124.62, 42.56.

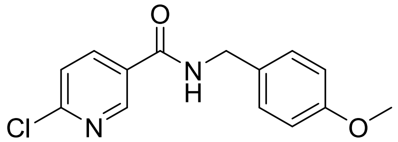

6-Chloro-N-(4-methoxy-benzyl)-nicotinamide(3j). White solid. ^{1}H NMR (400 MHz, DMSO-d_6) δ 9.23 (t, J = 5.9 Hz, 1H), 8.87 (d, J = 2.4 Hz, 1H), 8.27 (dd, J = 8.3, 2.5 Hz, 1H), 7.65 (d, J = 8.3 Hz, 1H), 7.31 – 7.20 (m, 2H), 6.94 – 6.86 (m, 2H), 4.43 (d, J = 5.8 Hz, 2H), 3.73 (s, 3H). ^{13}C NMR (101 MHz, DMSO-d_6) δ 164.03, 158.78, 153.01, 149.46, 139.09, 131.47, 129.74, 129.22, 124.60, 114.21, 55.53, 42.66.

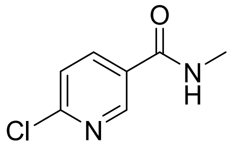

6-Chloro-N-methyl-nicotinamide(3k). White powder, ^{1}H NMR (400 MHz, Chloroform-*d*) δ 8.75 (d, *J* = 2.4 Hz, 1H), 8.10 (dd, *J* = 8.3, 2.5 Hz, 1H), 7.40 (d, *J* = 8.3 Hz, 1H), 6.81 (s, 1H), 3.02 (d, *J* = 4.7 Hz, 3H). ^{13}C NMR (101 MHz, Chloroform-*d*) δ 165.36, 154.08, 147.97, 138.01, 129.23, 124.39, 26.97.

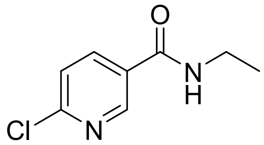

6-chloro-N-ethyl-3-Pyridinecarboxamide(3l). White power.
^{1}H NMR (400 MHz, Chloroform-*d*) δ 8.74 (d, *J* = 2.3 Hz, 1H), 8.09 (dd, *J* = 8.3, 2.3 Hz, 1H), 7.39 (d, *J* = 8.2 Hz, 1H), 6.72 (s, 1H), 3.55 – 3.43 (m, 2H), 1.25 (t, *J* = 7.2 Hz, 3H). ^{13}C NMR (101 MHz, Chloroform-*d*) δ 164.59, 154.00, 148.01, 138.01, 129.39, 124.33, 35.21, 14.72.

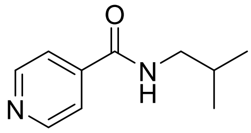

N-isobutyl-isonicotinamide(3m). White solid. ^{1}H NMR (400 MHz, DMSO-d_6) δ 8.80 – 8.66 (m, 3H), 7.79 – 7.69 (m, 2H), 3.11 (t, *J* = 6.4 Hz, 2H), 1.85 (dq, *J* = 13.5, 6.7 Hz, 1H), 0.90 (d, *J* = 6.7 Hz, 6H). ^{13}C NMR (101 MHz, DMSO-d_6) δ 164.77, 150.21, 141.69, 121.28, 46.78, 28.05, 20.20.

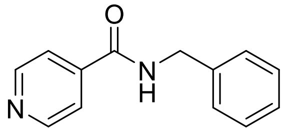

N-benzyl-isonicotinamide (3n). White solid. ^{1}H NMR (400 MHz, DMSO-d_6) δ 9.40 – 9.33 (m, 1H), 8.75 (d, *J* = 5.0 Hz, 2H), 7.82 (d, *J* = 5.1 Hz, 2H), 7.35 (d, *J* = 4.5 Hz, 4H), 7.27 (p, *J* = 5.8, 4.4 Hz, 1H), 4.52 (d, *J* = 6.1 Hz, 2H). ^{13}C NMR (101 MHz, DMSO-d6) δ 164.69, 150.29, 141.24, 139.11, 128.36, 127.29, 126.91, 121.28, 42.72.

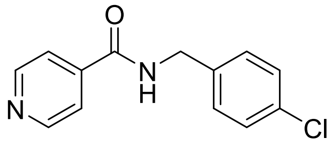

N-[(4-chlorophenyl) methyl]-4-Pyridinecarboxamide(3o). White solid. ^{1}H NMR (400 MHz, DMSO-d_6) δ 9.38 (t, *J* = 6.0 Hz, 1H), 8.81 – 8.68 (m, 2H), 7.86 – 7.77 (m, 2H), 7.45 – 7.32 (m, 4H), 4.50 (d, *J* = 5.9 Hz, 2H). ^{13}C NMR (101 MHz, DMSO-d_6) δ 164.74, 150.31, 141.09, 138.16, 131.46, 129.19, 128.31, 121.26, 42.09.

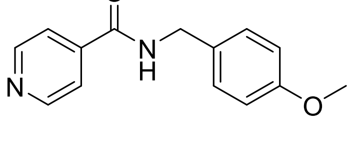

N-[(4-methoxyphenyl) methyl]- 4-Pyridinecarboxamide(3p). White solid. ^{1}H NMR (400 MHz, DMSO-d_6) δ 9.29 (t, *J* = 6.1 Hz, 1H), 8.74 (d, *J* = 5.2 Hz, 2H), 7.80 (d, *J* = 5.1 Hz, 2H), 7.27 (d, *J* = 8.1 Hz,

2H), 6.91 (d, J = 8.0 Hz, 2H), 4.44 (d, J = 5.8 Hz, 2H), 3.74 (d, J = 3.5 Hz, 3H). ^{13}C NMR (101 MHz, DMSO-d_6) δ 164.54, 158.31, 150.27, 141.32, 131.06, 128.71, 121.27, 113.75, 55.07, 42.20.

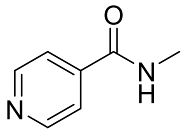

1-methylisonicotinamide(3q). White power. ^{1}H NMR (400 MHz, DMSO-d_6) δ 8.77 – 8.69 (m, 3H), 7.77 – 7.71 (m, 2H), 2.81 (d, J = 4.6 Hz, 3H). ^{13}C NMR (101 MHz, DMSO-d_6) δ 165.46, 150.69, 141.83, 121.57, 26.71.

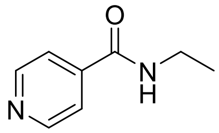

N-Ethylisonicotinamide: (3r). White power. ^{1}H NMR (400 MHz, DMSO-d_6) δ 8.77 (t, J = 5.5 Hz, 1H), 8.75 – 8.69 (m, 2H), 7.78 – 7.72 (m, 2H), 3.35 – 3.25 (m, 2H), 1.13 (t, J = 7.2 Hz, 3H). ^{13}C NMR (101 MHz, DMSO-d_6) δ 164.78, 150.66, 141.99, 121.64, 34.63, 15.04.

3a

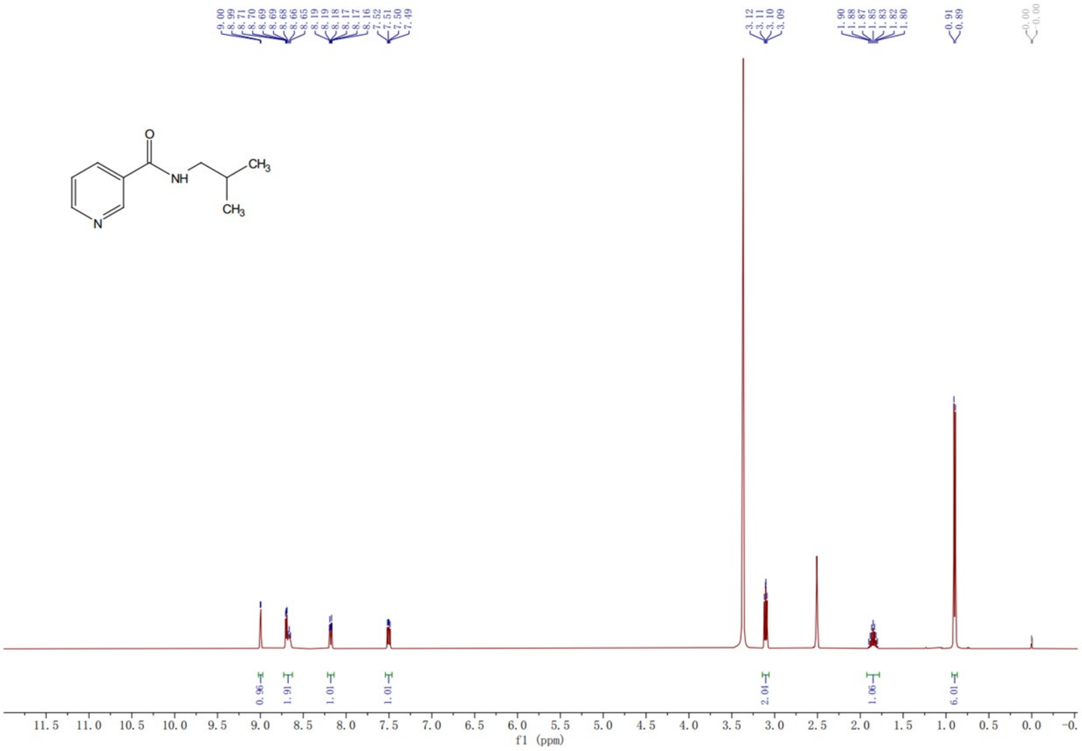
9.00
8.99
8.71
8.70
8.69
8.69
8.68
8.66
8.65
8.19
8.19
8.18
8.17
8.17
8.16
7.52
7.51
7.50
7.49
3.12
3.11
3.10
3.09
1.90
1.88
1.87
1.85
1.83
1.82
1.80
0.91
0.89
0.00
0.00
O
NH
CH3
CH3
N
0.96
1.91
1.01
1.01
2.04
1.06
6.01
f1 (ppm)

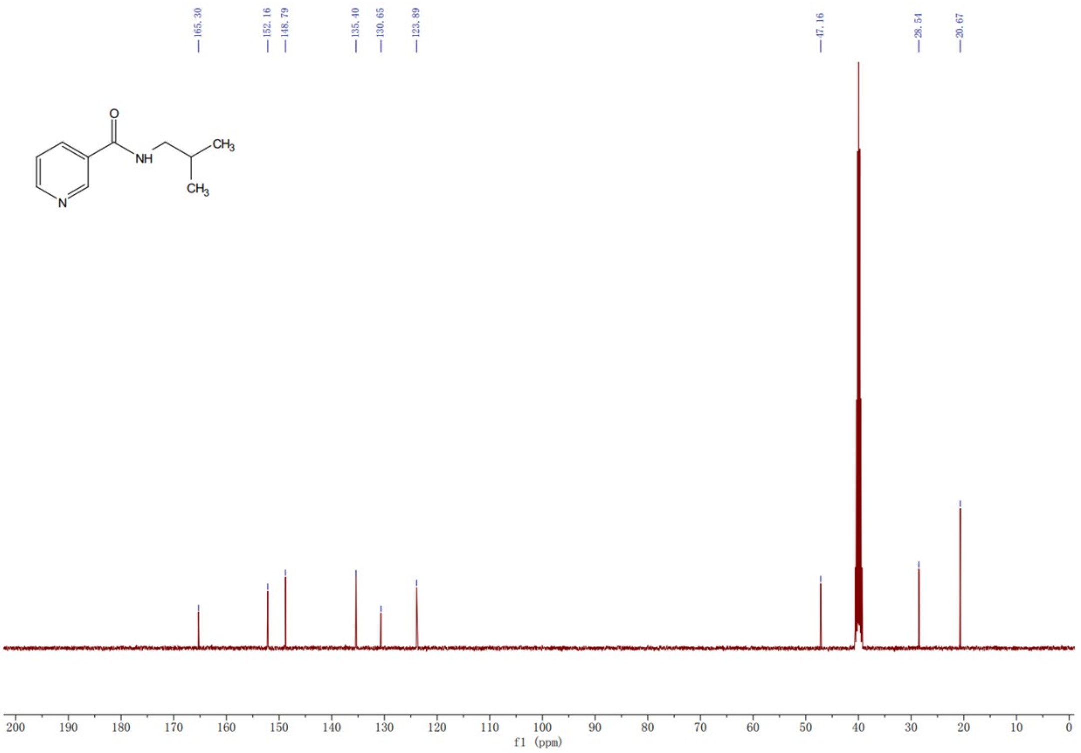
165.30
152.16
148.79
135.40
130.65
123.89
47.16
28.54
20.67
O
NH
CH3
CH3
N
f1 (ppm)

3b

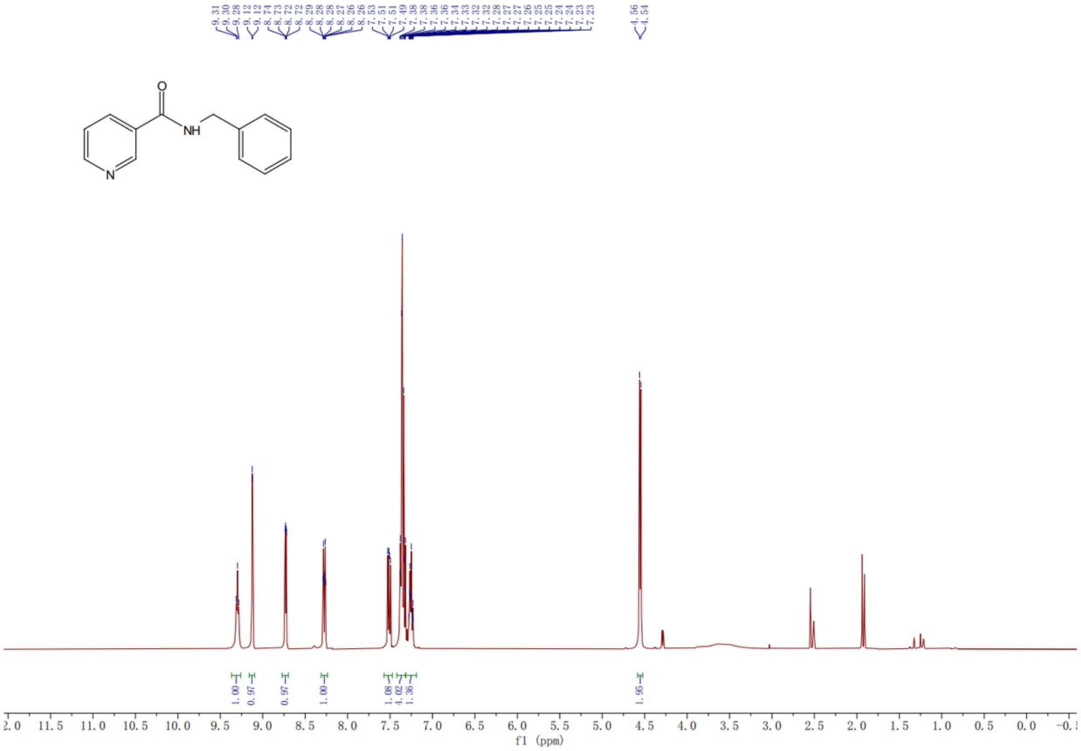

9.31
9.30
9.28
9.12
9.12
8.74
8.73
8.72
8.72
8.29
8.28
8.28
8.27
8.26
8.26
7.53
7.51
7.51
7.49
7.38
7.38
7.36
7.36
7.34
7.33
7.32
7.32
7.28
7.27
7.27
7.26
7.25
7.25
7.24
7.24
7.23
7.23
4.56
4.54
O
NH
N
1.00
0.97
0.97
1.00
1.08
4.02
1.36
1.95
f1 (ppm)

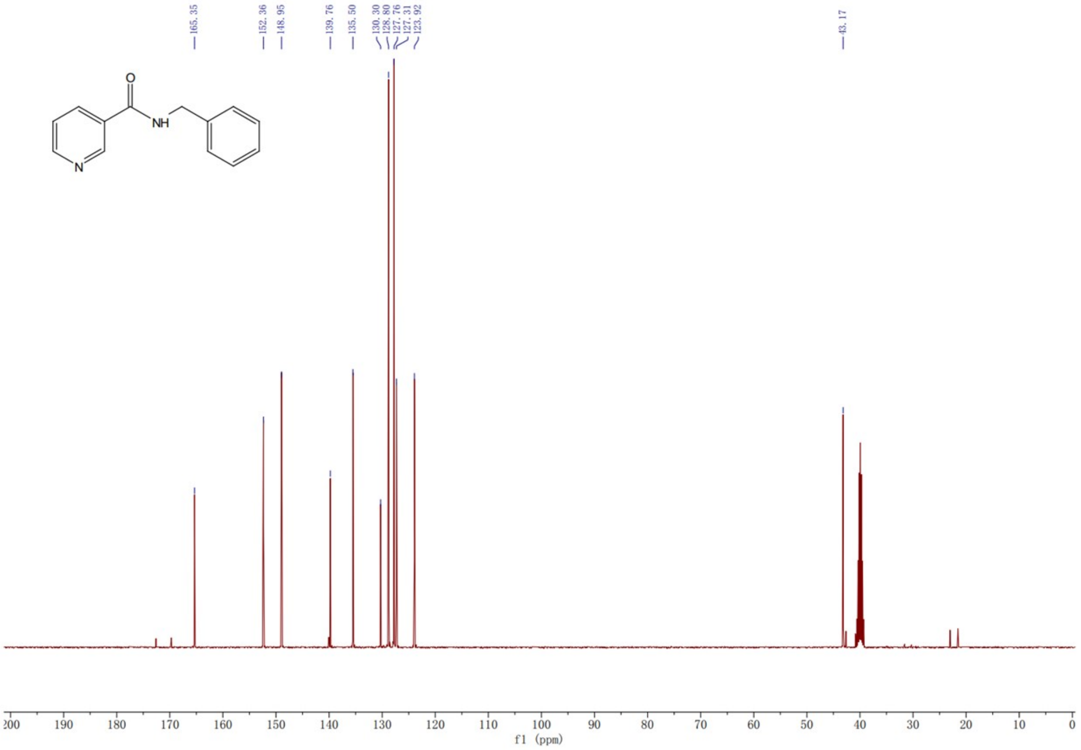

165.35
152.36
148.95
139.76
135.50
130.30
128.80
127.76
127.31
123.92
43.17
O
NH
N
f1 (ppm)

3c

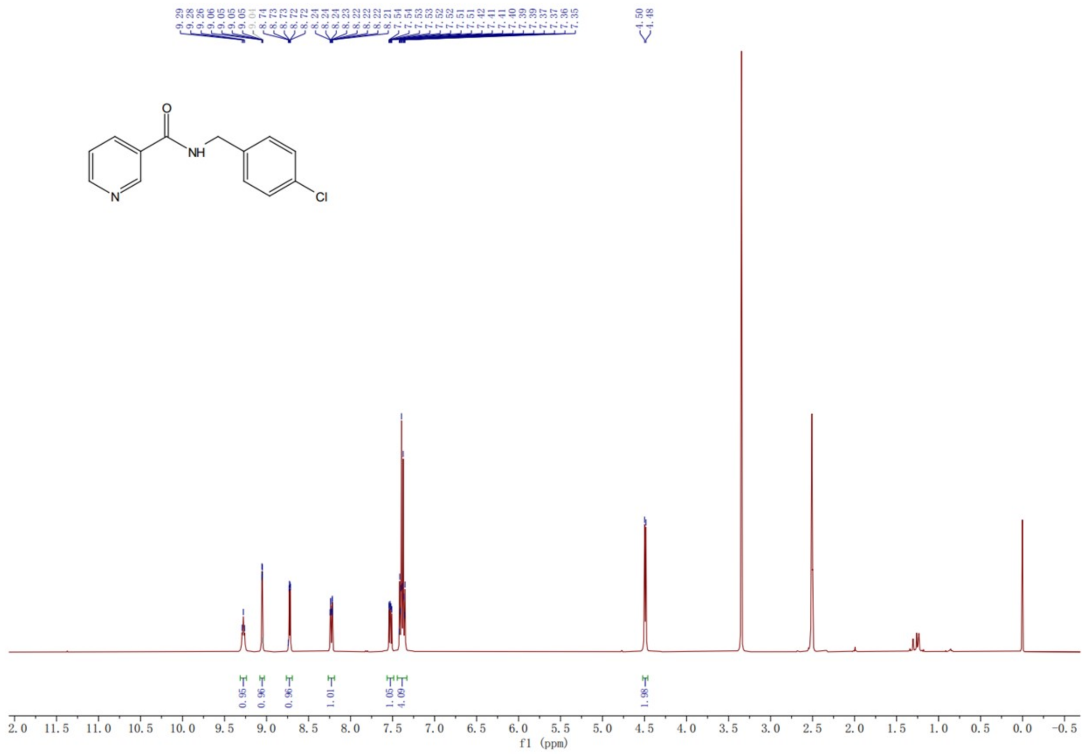

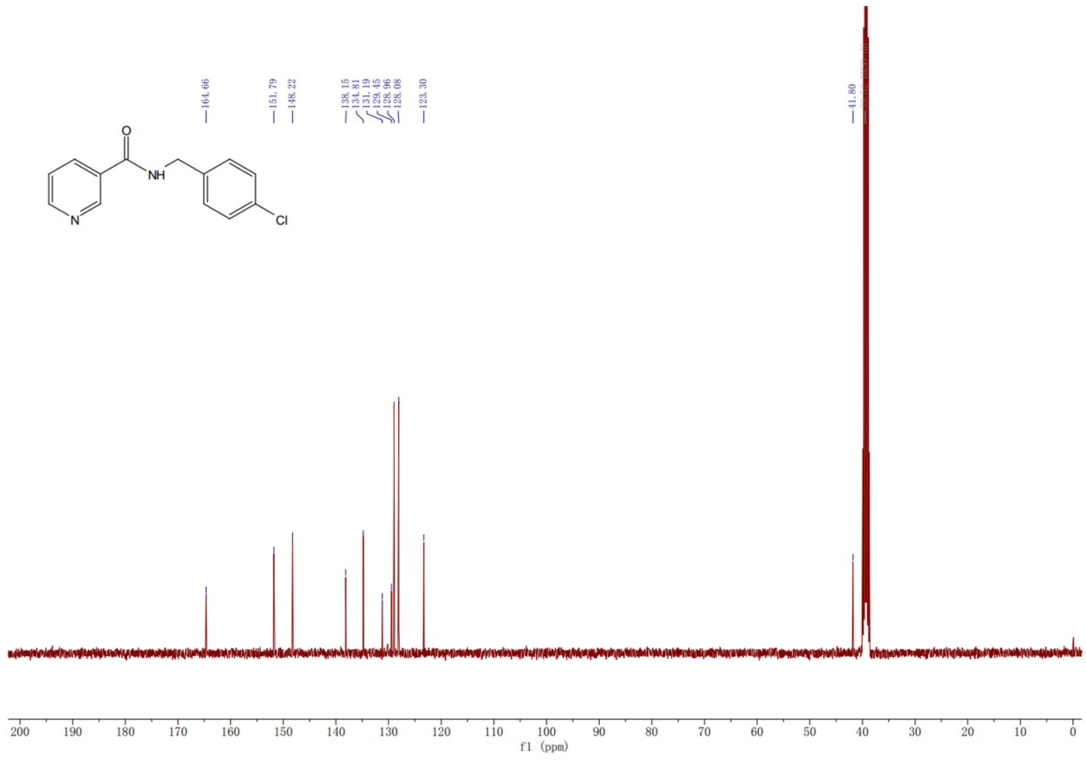

3d

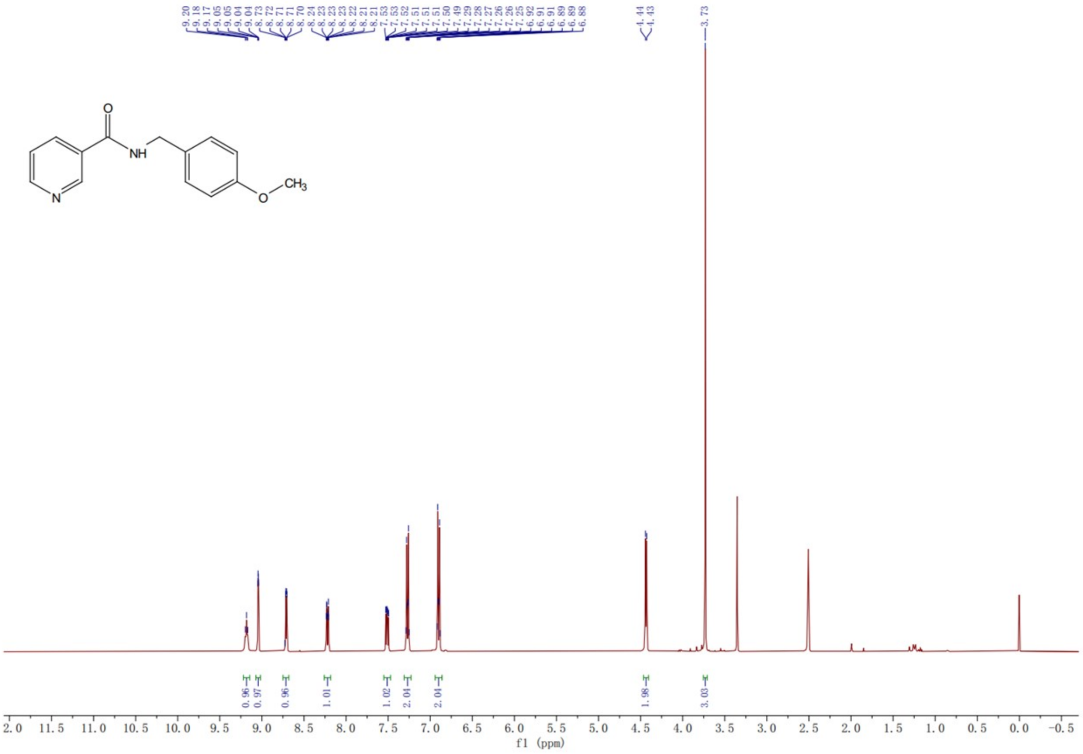
9.20 9.18 9.17 9.05 9.05 9.04 9.04 8.73 8.72 8.71 8.71 8.70 8.24 8.23 8.23 8.23 8.22 8.21 8.21 8.21 7.53 7.53 7.52 7.51 7.51 7.51 7.50 7.49 7.29 7.28 7.27 7.26 7.25 6.92 6.91 6.89 6.89 6.88
4.44 4.43
3.73
O
NH
N
O
CH3
0.96 0.97 0.96 1.01 1.02 2.04 2.04 1.98 3.03
f1 (ppm)

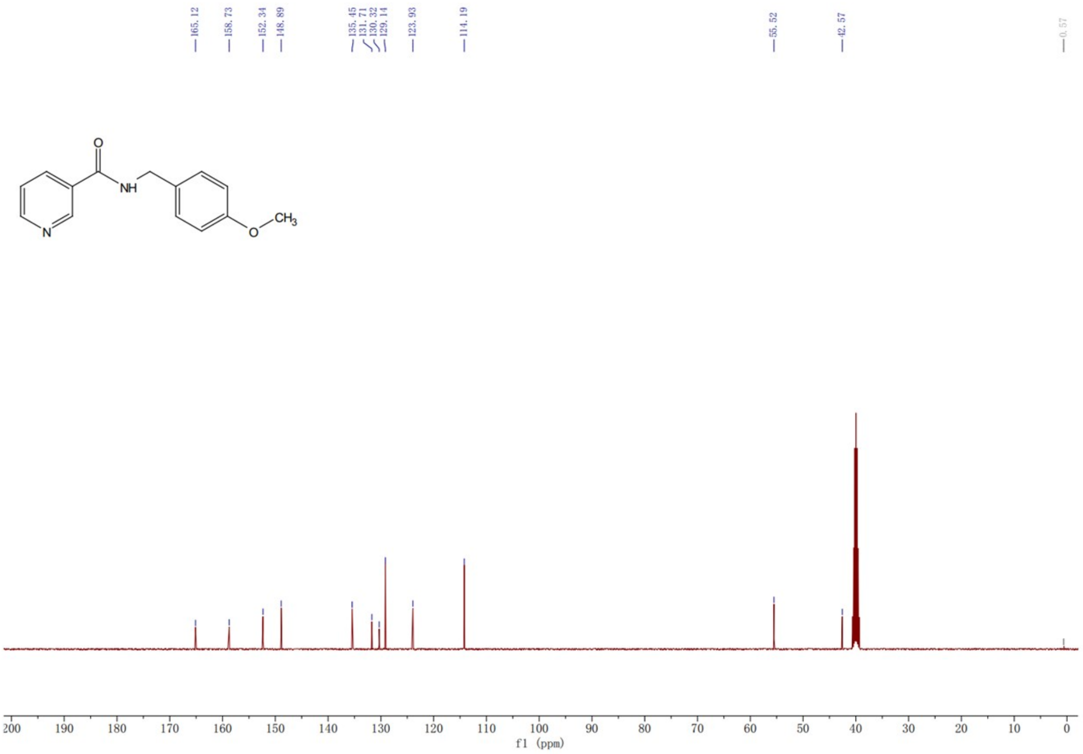
165.12
158.73
152.34
148.89
135.45 131.71 130.32 129.14
123.93
114.19
55.52
42.57
0.57
O
NH
N
O
CH3
f1 (ppm)

3e

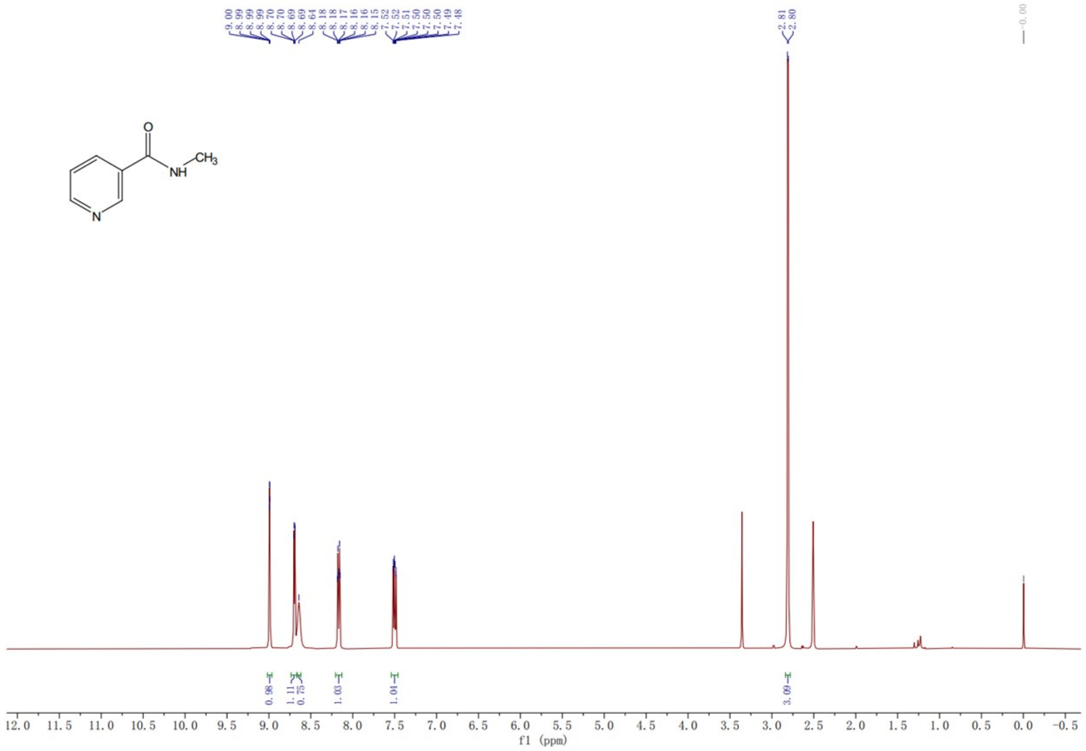

9.00
8.99
8.99
8.99
8.70
8.70
8.69
8.69
8.64
8.18
8.18
8.17
8.16
8.16
8.15
7.52
7.52
7.51
7.50
7.50
7.50
7.49
7.48
2.81
2.80
0.00
0.98
1.11
0.75
1.03
1.04
3.09
f1 (ppm)

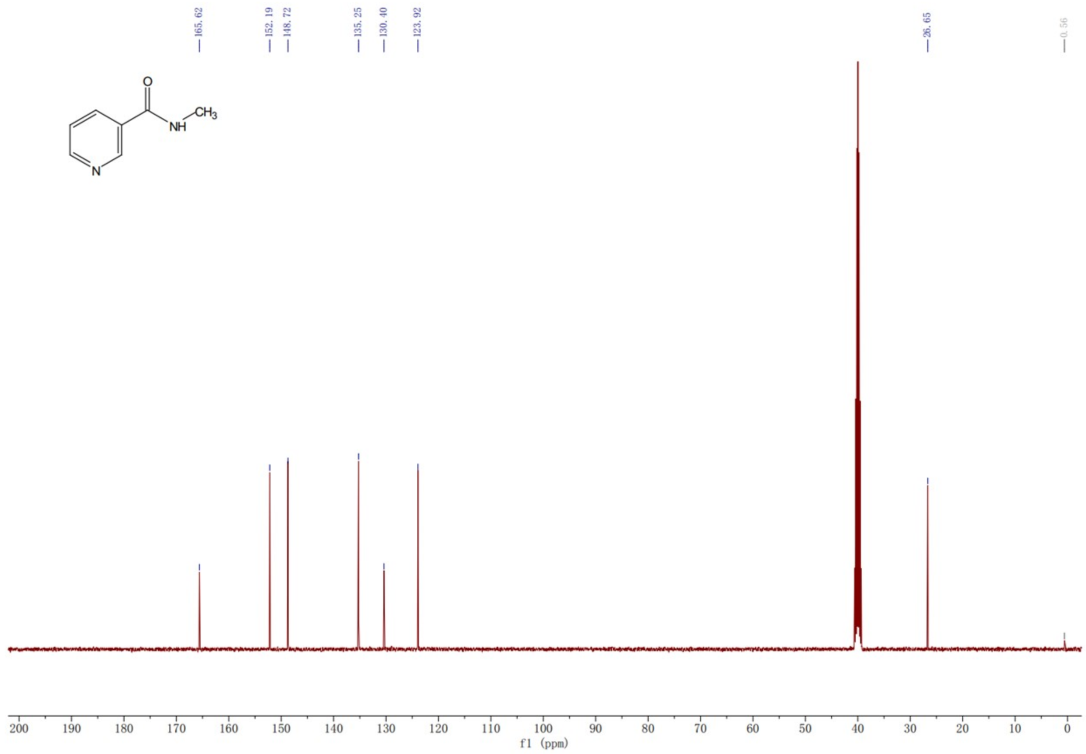

165.62
152.19
148.72
135.25
130.40
123.92
26.65
0.56
f1 (ppm)

3f

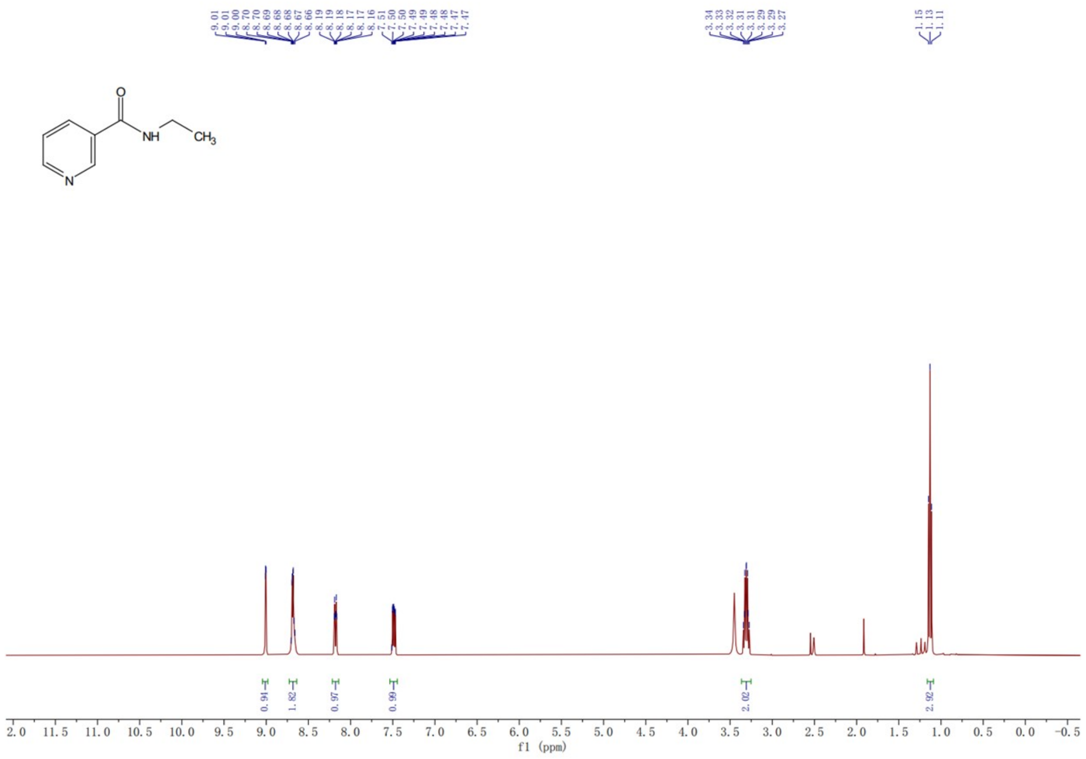

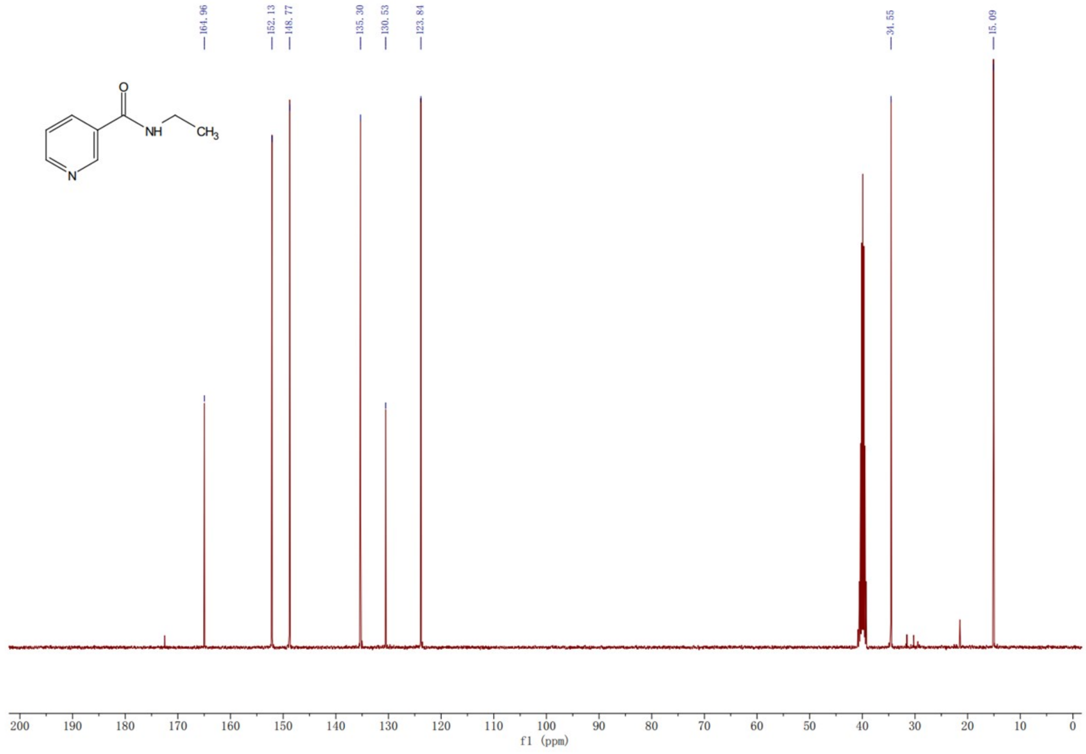

3g

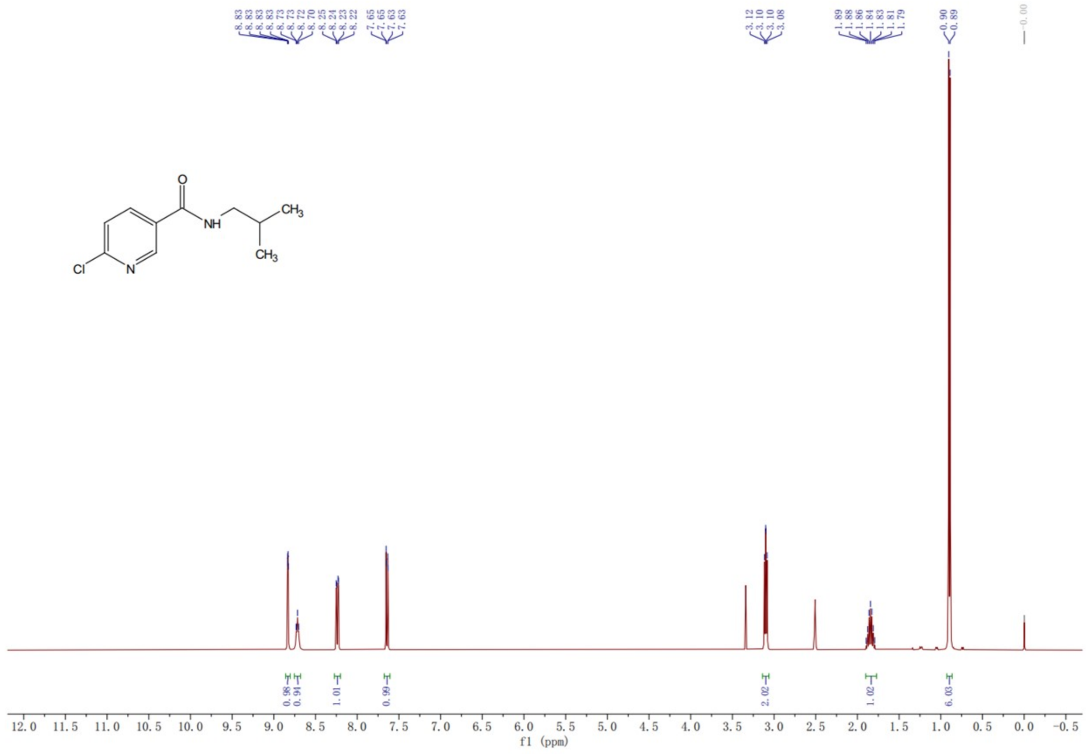

8.83
8.83
8.83
8.83
8.73
8.72
8.70
8.25
8.24
8.23
8.22
7.65
7.65
7.63
7.63
3.12
3.10
3.10
3.08
1.89
1.88
1.86
1.84
1.83
1.81
1.79
0.90
0.89
0.00
0.98
0.94
1.01
0.99
2.02
1.02
6.03
f1 (ppm)

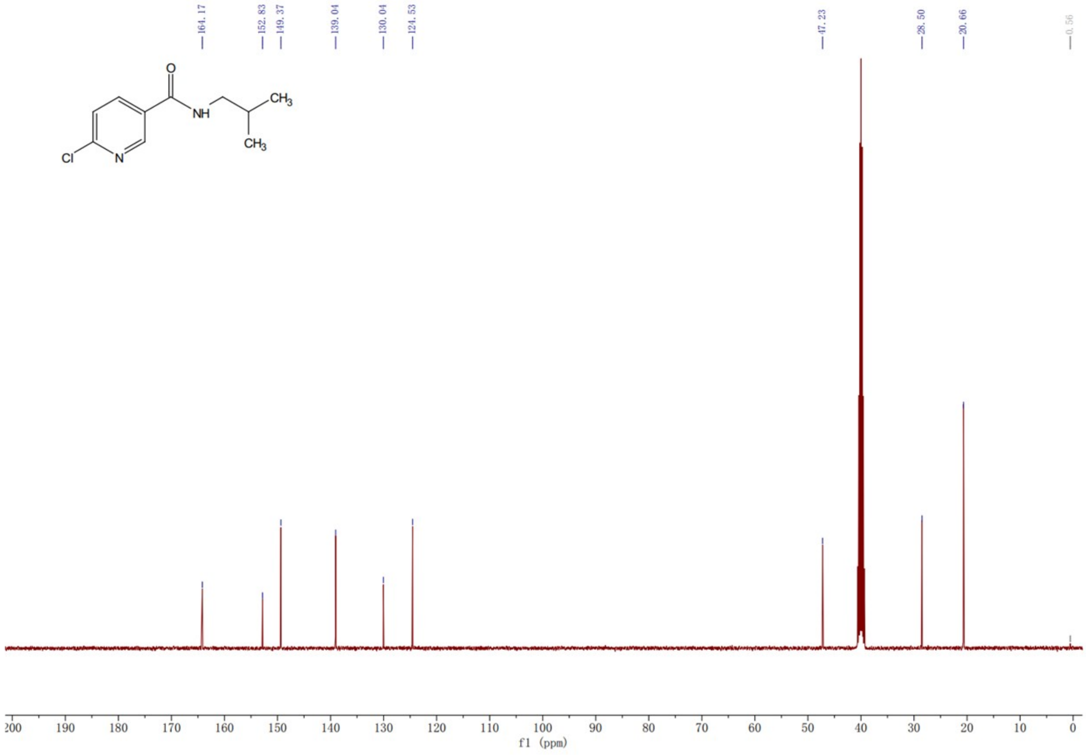

164.17
152.83
149.37
139.04
130.04
124.53
47.23
28.50
20.66
0.56
f1 (ppm)

3h

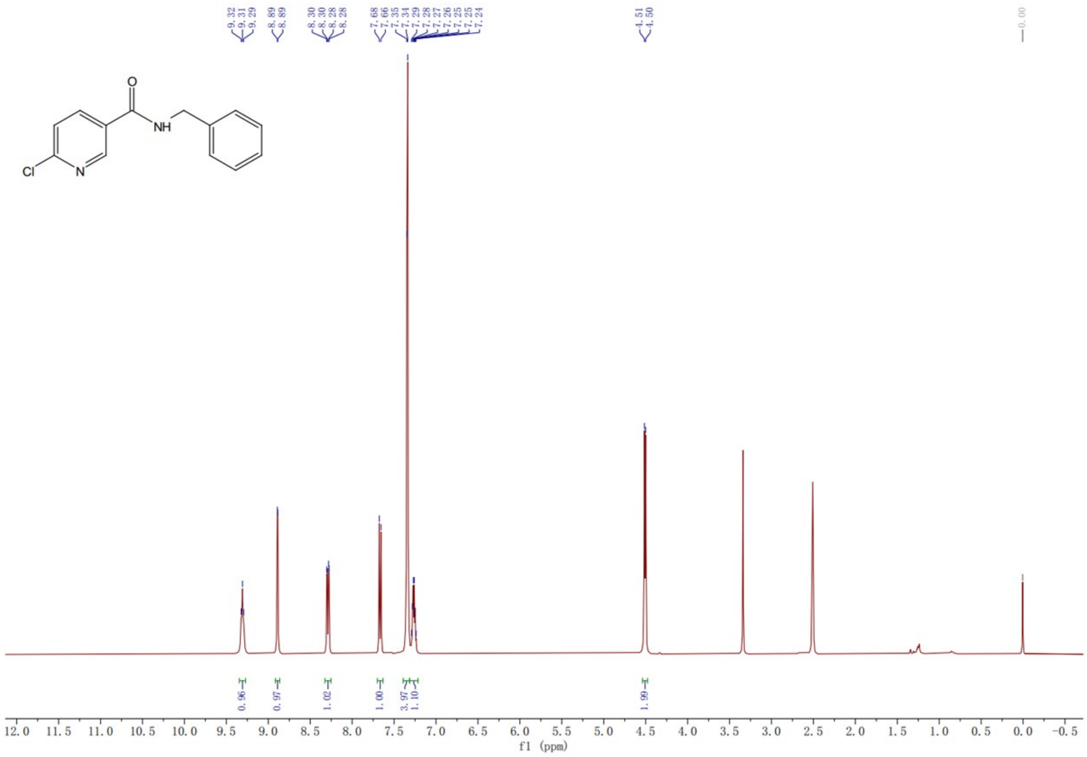

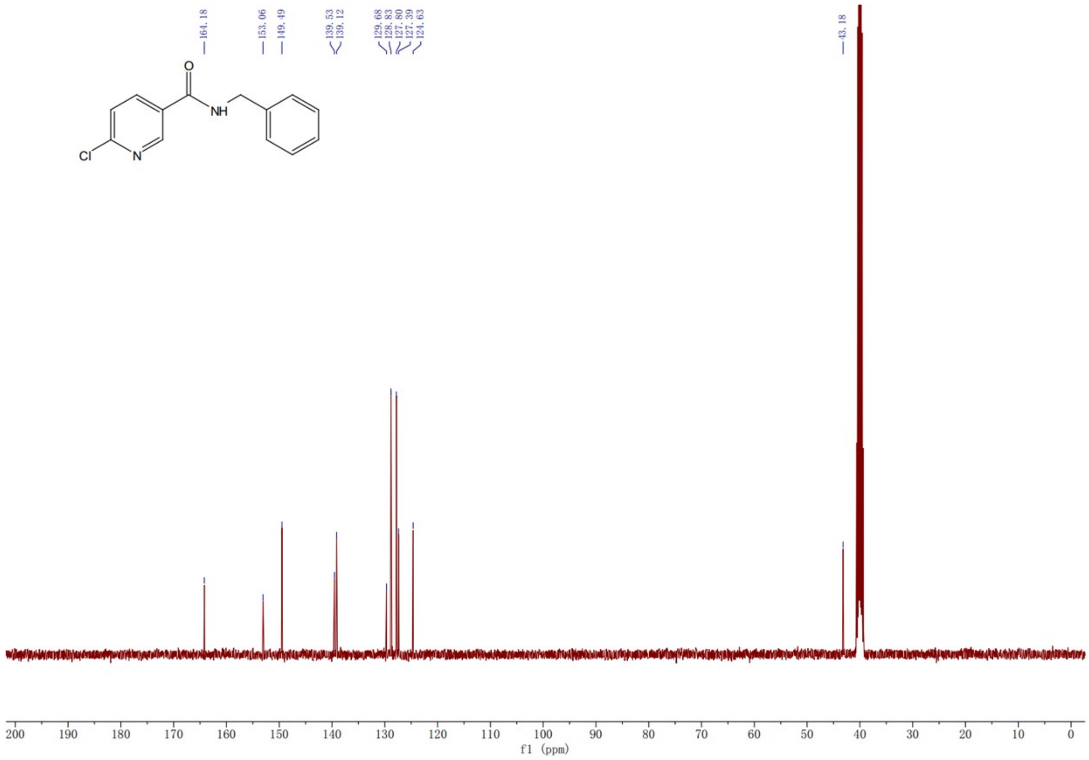

3i

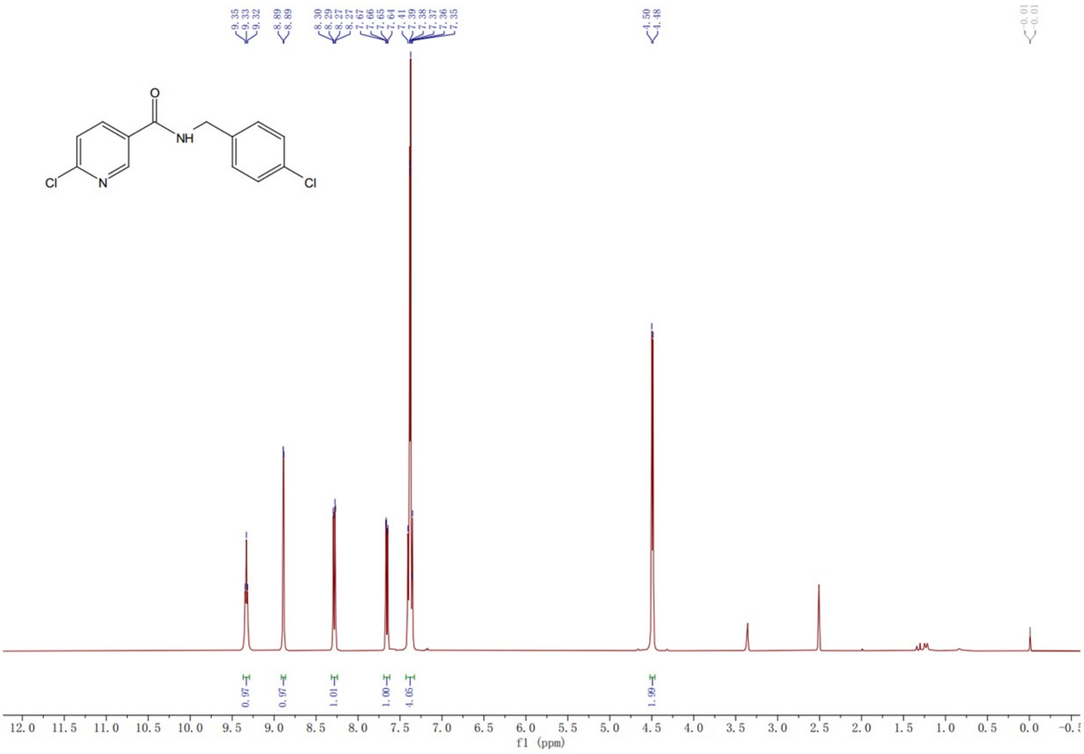
9.35
9.33
9.32
8.89
8.89
8.30
8.29
8.27
8.27
7.67
7.66
7.65
7.64
7.41
7.39
7.38
7.37
7.36
7.35
4.50
4.48
0.01
0.01
O
NH
Cl
N
Cl
0.97
0.97
1.01
1.00
4.05
1.99
f1 (ppm)

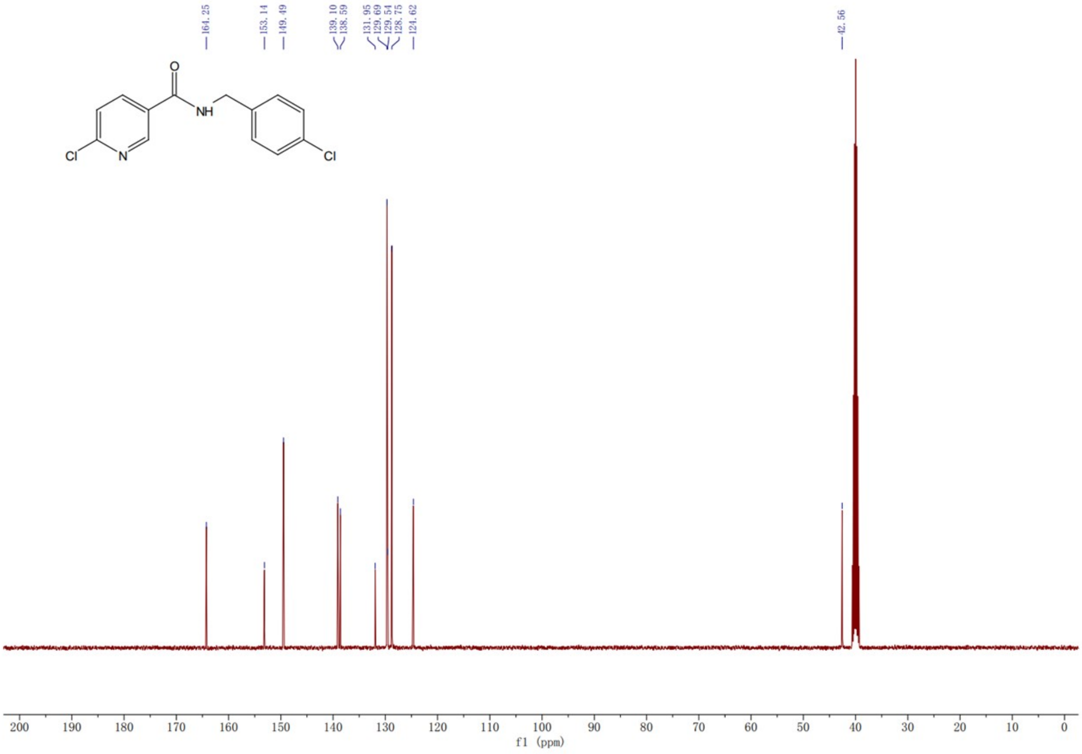
164.25
153.14
149.49
139.10
138.59
131.95
129.69
129.54
128.75
124.62
42.56
O
NH
Cl
N
Cl
f1 (ppm)

3j

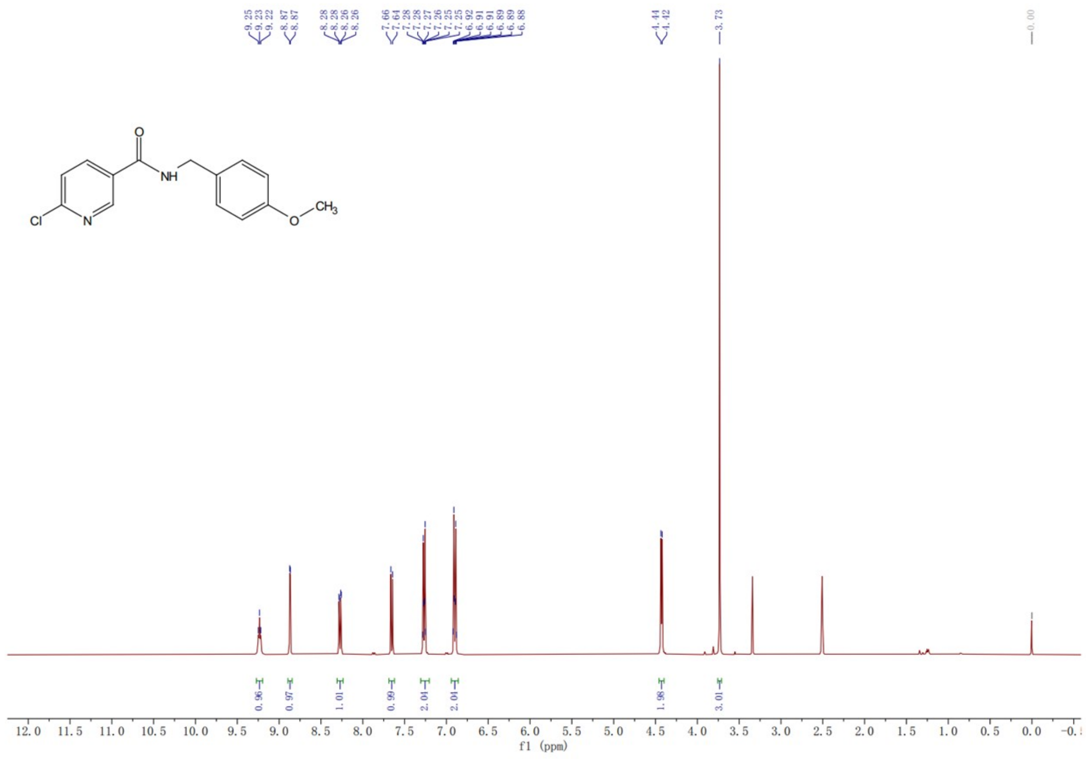

O
NH
Cl
N
O
CH3
f1 (ppm)

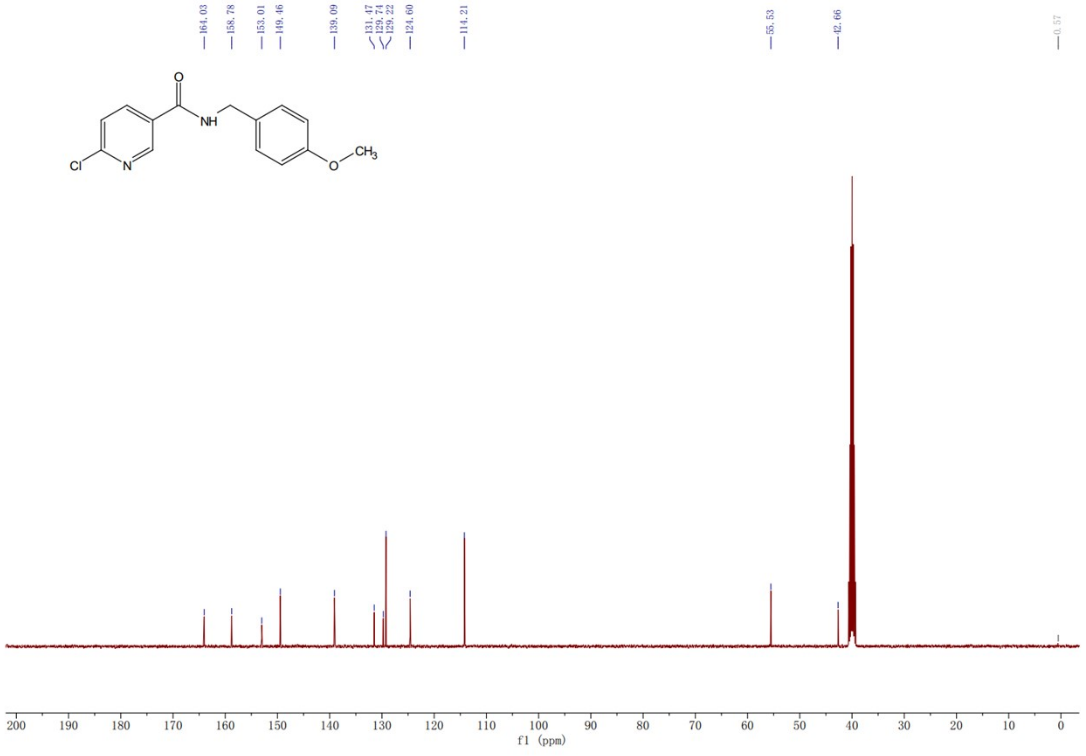

164.03
158.78
153.01
149.46
139.09
131.47
129.74
129.22
124.60
114.21
55.53
42.66
O
NH
Cl
N
O
CH3
f1 (ppm)

3k

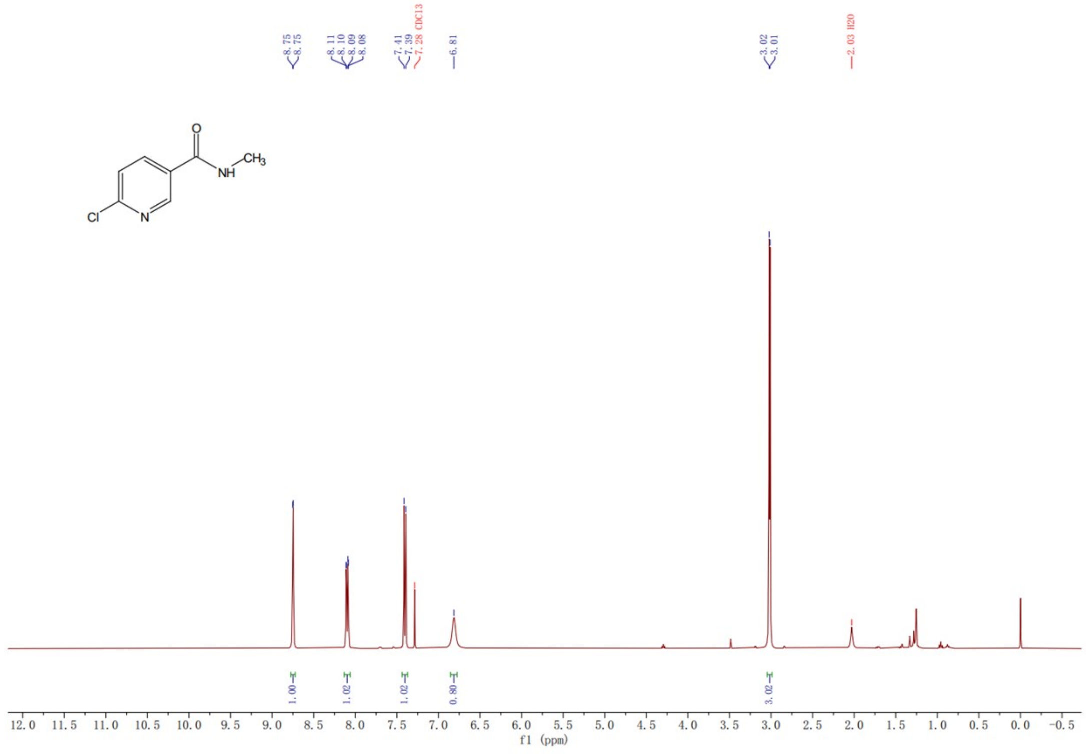

8.75
8.75
8.11
8.10
8.09
8.08
7.41
7.39
7.28 CDCl3
6.81
3.02
3.01
2.03 H2O
O
NH
CH3
Cl
N
1.00
1.02
1.02
0.80
3.02
12.0 11.5 11.0 10.5 10.0 9.5 9.0 8.5 8.0 7.5 7.0 6.5 6.0 5.5 5.0 4.5 4.0 3.5 3.0 2.5 2.0 1.5 1.0 0.5 0.0 -0.5
f1 (ppm)

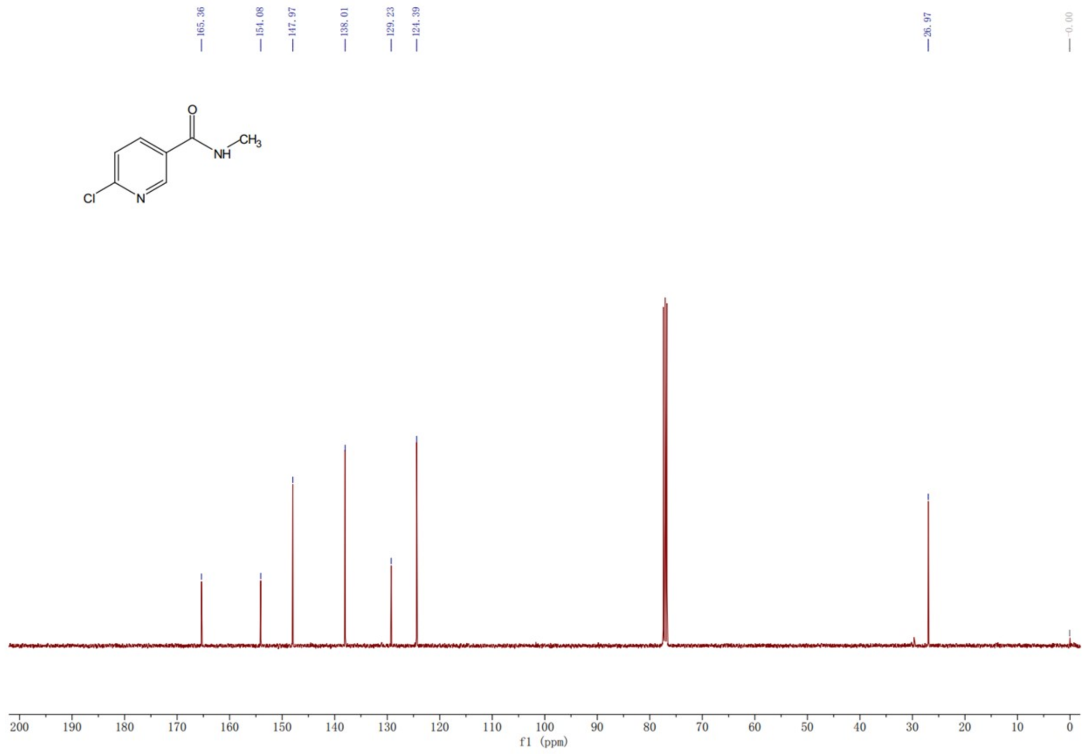

165.36
154.08
147.97
138.01
129.23
124.39
26.97
0.00
O
NH
CH3
Cl
N
200 190 180 170 160 150 140 130 120 110 100 90 80 70 60 50 40 30 20 10 0
f1 (ppm)

31

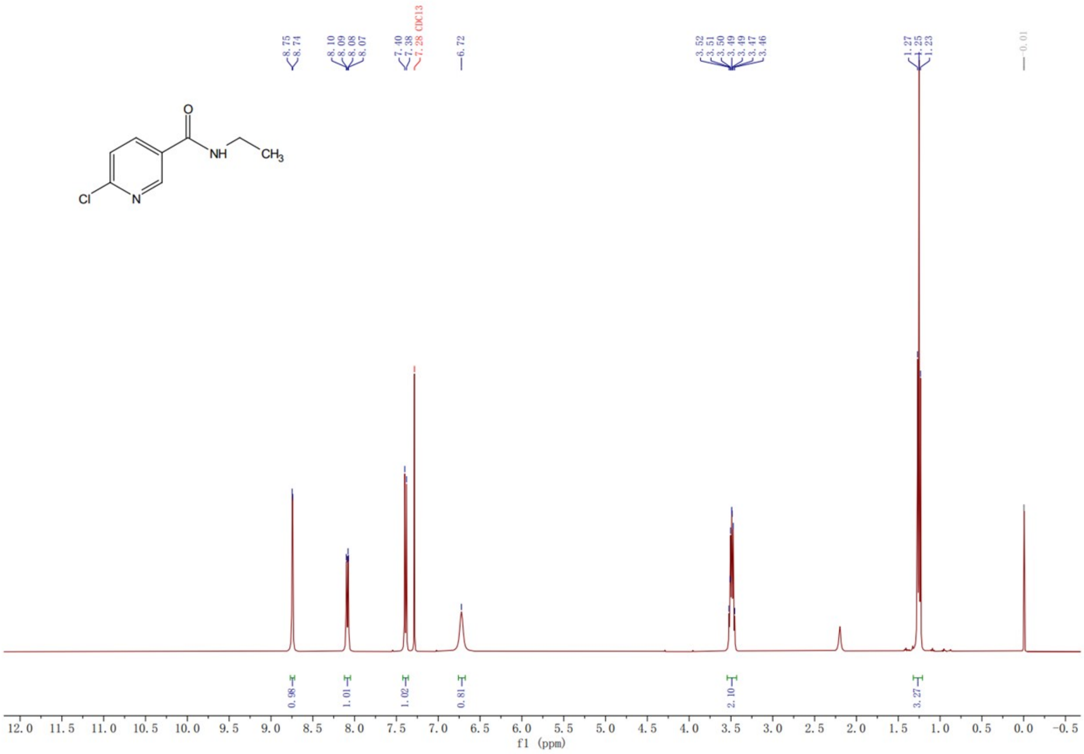
8.75
8.74
8.10
8.09
8.08
8.07
7.40
7.38
7.28 CDCl3
6.72
3.52
3.51
3.50
3.49
3.49
3.47
3.46
1.27
1.25
1.23
0.01
0.98
1.01
1.02
0.81
2.10
3.27
NH
CH3
Cl
N
O
f1 (ppm)

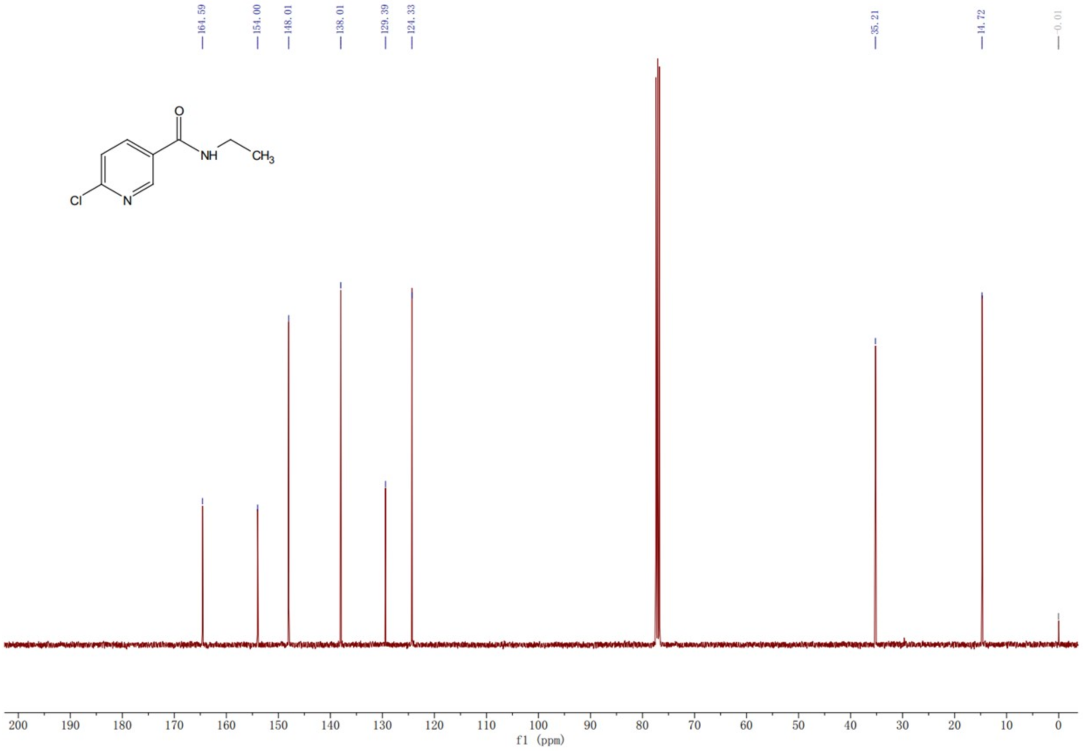
164.59
154.00
148.01
138.01
129.39
124.33
35.21
14.72
-0.01
NH
CH3
Cl
N
O
f1 (ppm)

3m

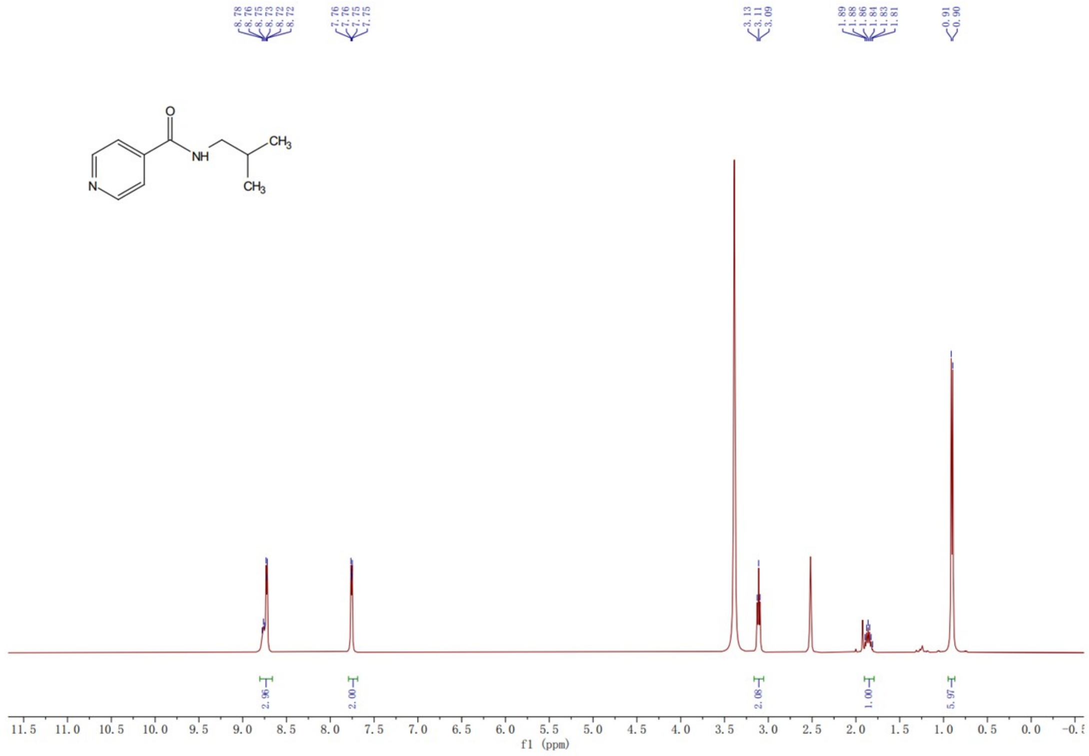

8.78
8.76
8.75
8.73
8.72
8.72
7.76
7.76
7.75
7.75
3.13
3.11
3.09
1.89
1.88
1.86
1.84
1.83
1.81
0.91
0.90
O
NH
CH3
N
CH3
2.96
2.00
2.08
1.00
5.97
f1 (ppm)

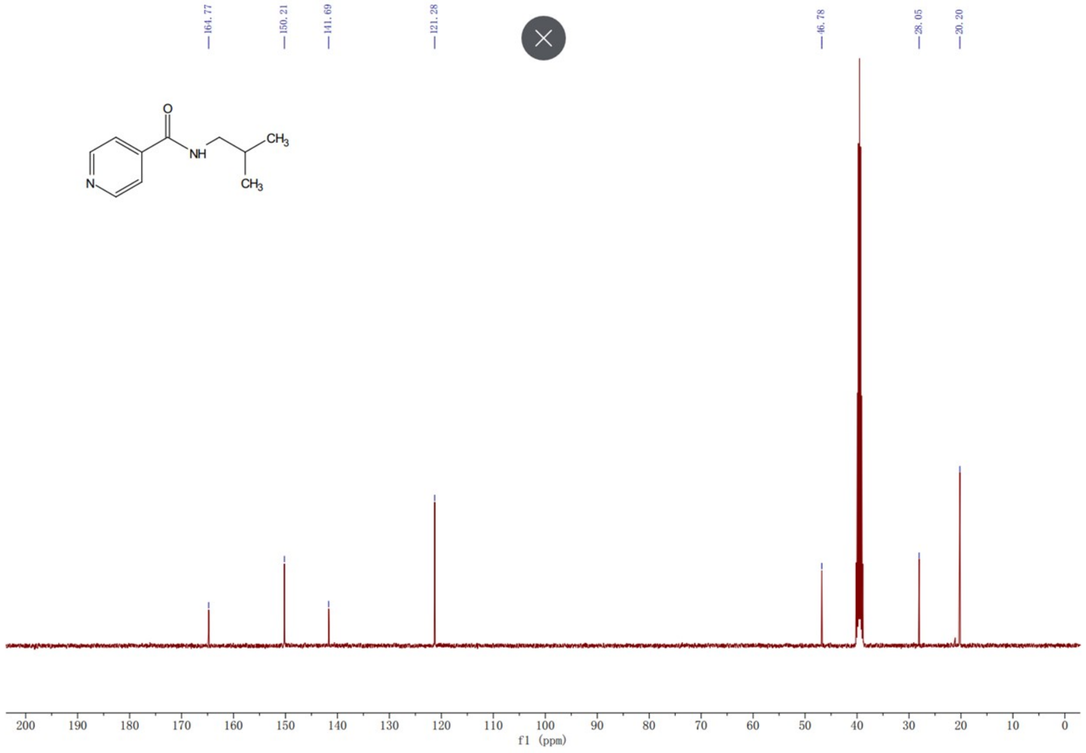

164.77
150.21
141.69
121.28
46.78
28.05
20.20
O
NH
CH3
N
CH3
f1 (ppm)

3n

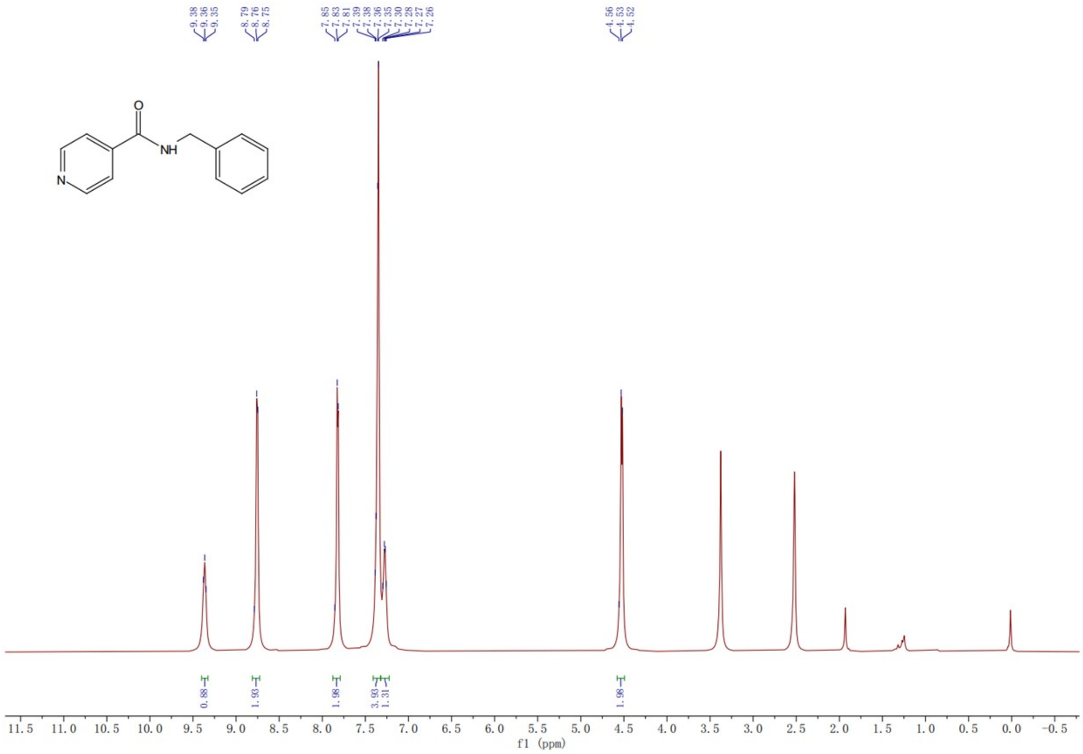
9.38
9.36
9.35
8.79
8.76
8.75
7.85
7.83
7.81
7.39
7.38
7.36
7.35
7.30
7.28
7.27
7.26
4.56
4.53
4.52
O
NH
N
0.88
1.93
1.98
3.93
1.31
1.98
f1 (ppm)

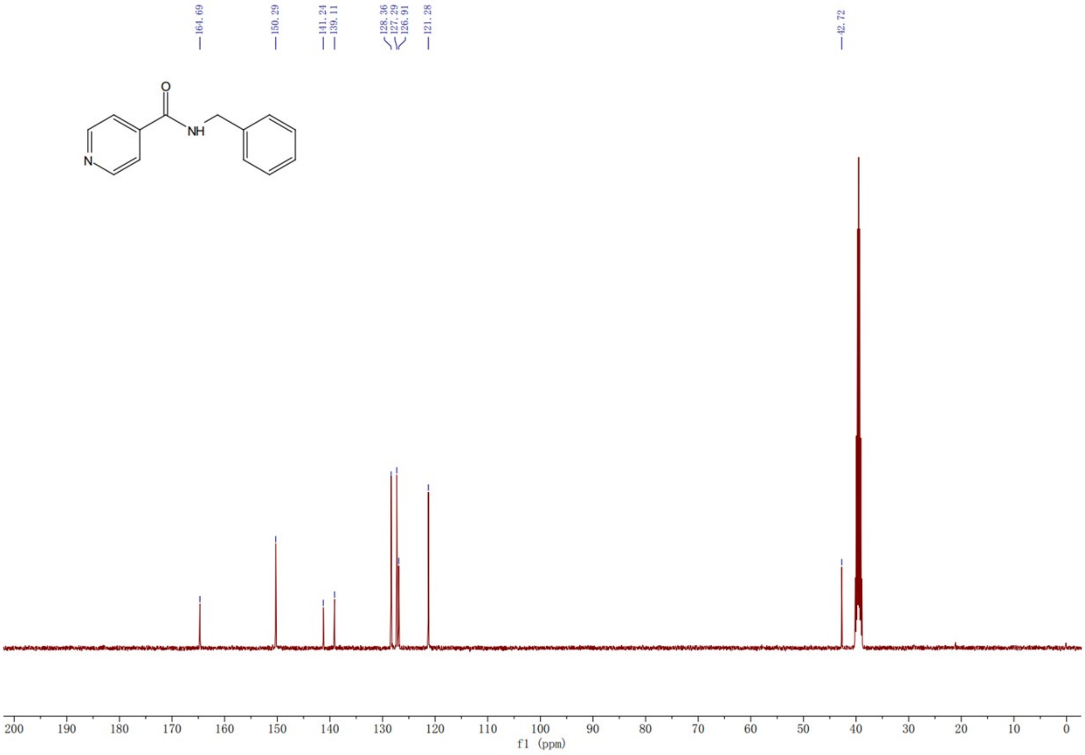
164.69
150.29
141.24
139.11
128.36
127.29
126.91
121.28
42.72
O
NH
N
f1 (ppm)

3o

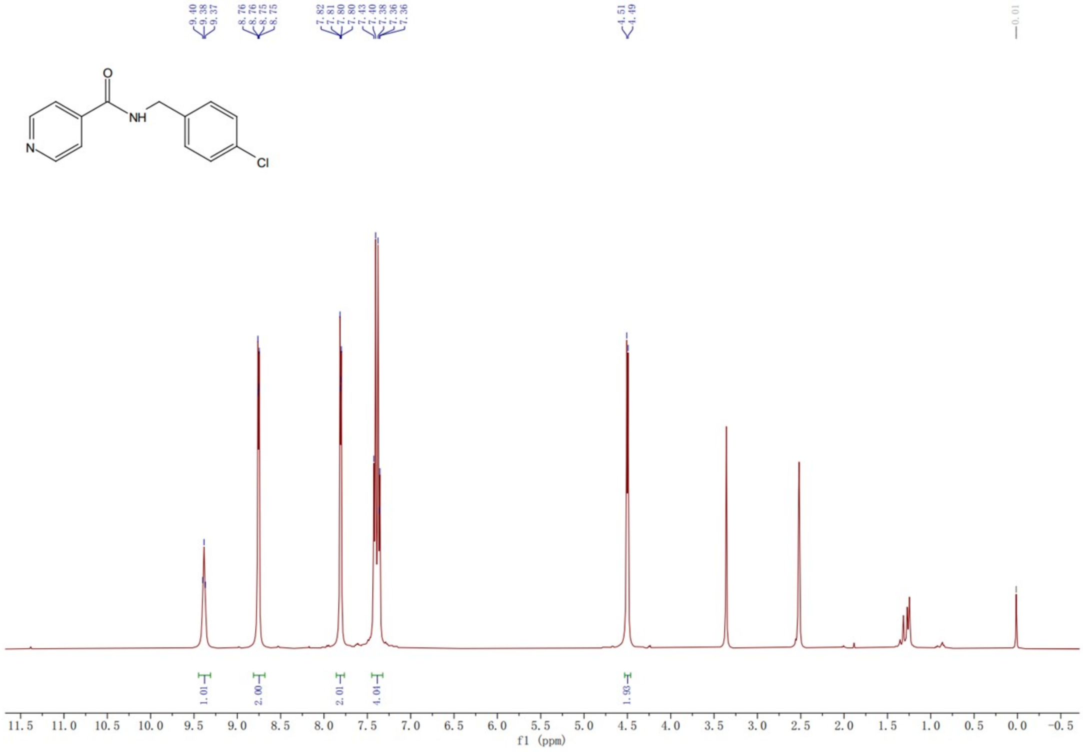

9.40
9.38
9.37
8.76
8.76
8.75
8.75
7.82
7.81
7.80
7.80
7.43
7.40
7.38
7.36
7.36
4.51
4.49
0.01
NH
O
N
Cl
1.01
2.00
2.01
4.01
1.93
f1 (ppm)

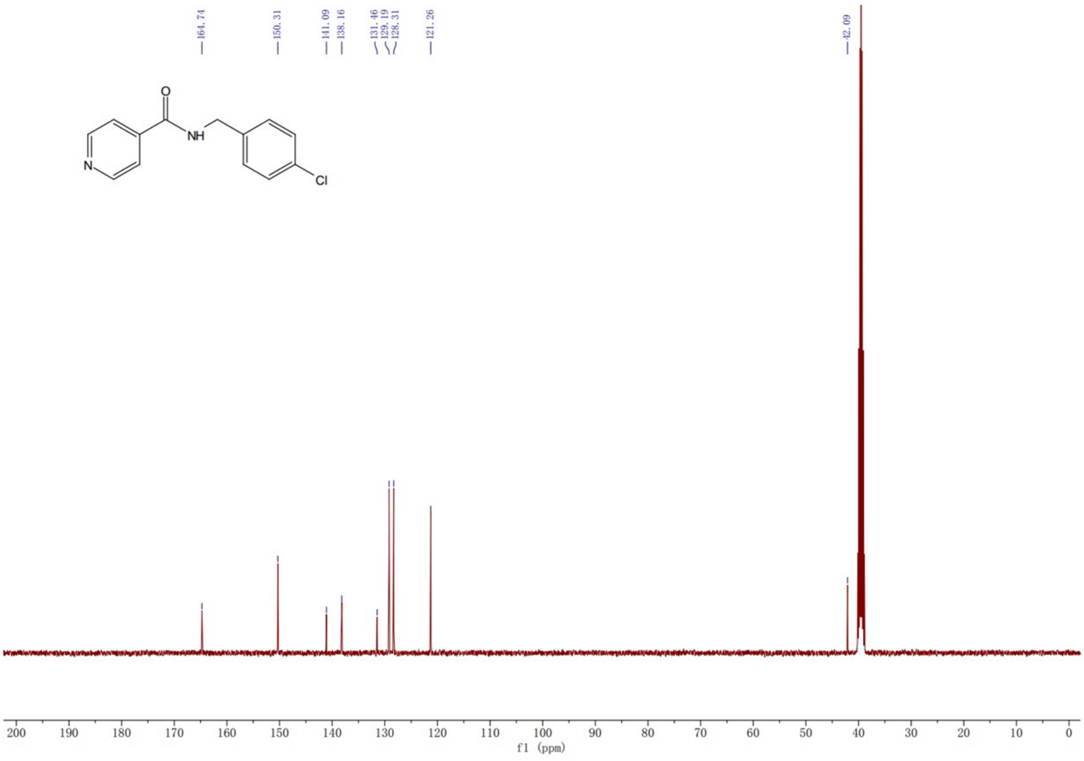

164.74
150.31
141.09
138.16
131.46
129.19
128.31
121.26
42.09
NH
O
N
Cl
f1 (ppm)

3p

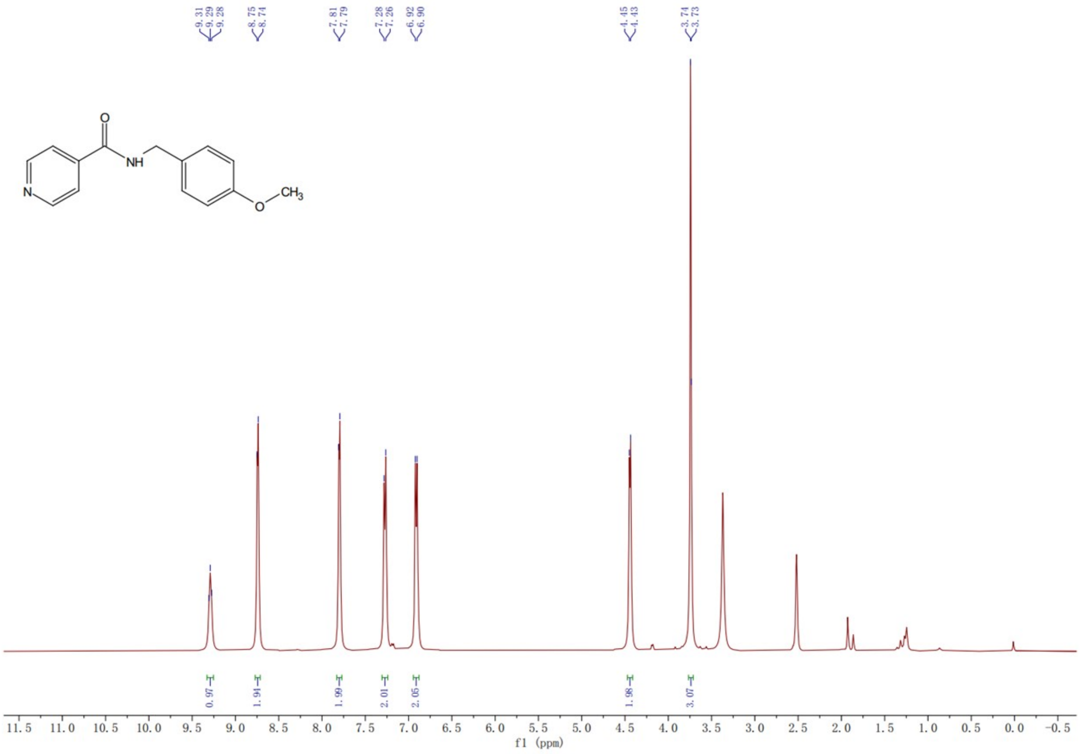

9.31
9.29
9.28
8.75
8.74
7.81
7.79
7.28
7.26
6.92
6.90
4.45
4.43
3.74
3.73
O
NH
N
O
CH3
0.97
1.94
1.99
2.01
2.05
1.98
3.07
11.5 11.0 10.5 10.0 9.5 9.0 8.5 8.0 7.5 7.0 6.5 6.0 5.5 5.0 4.5 4.0 3.5 3.0 2.5 2.0 1.5 1.0 0.5 0.0 -0.5
f1 (ppm)

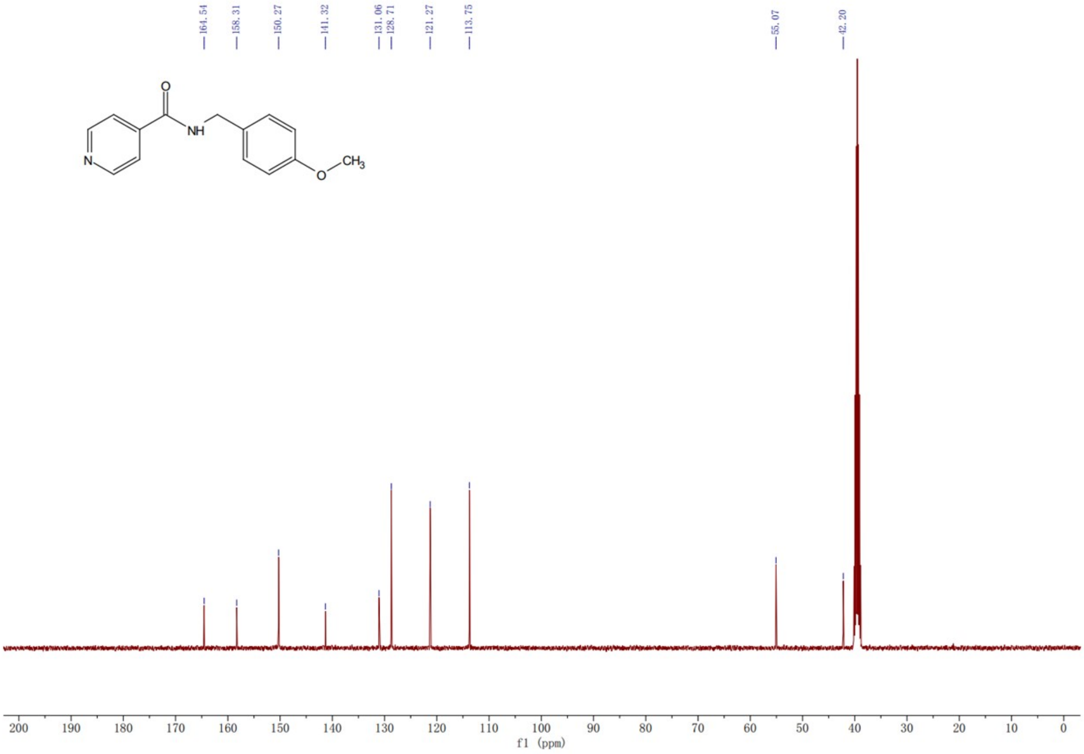

164.54
158.31
150.27
141.32
131.06
128.71
121.27
113.75
55.07
42.20
O
NH
N
O
CH3
200 190 180 170 160 150 140 130 120 110 100 90 80 70 60 50 40 30 20 10 0
f1 (ppm)

3q

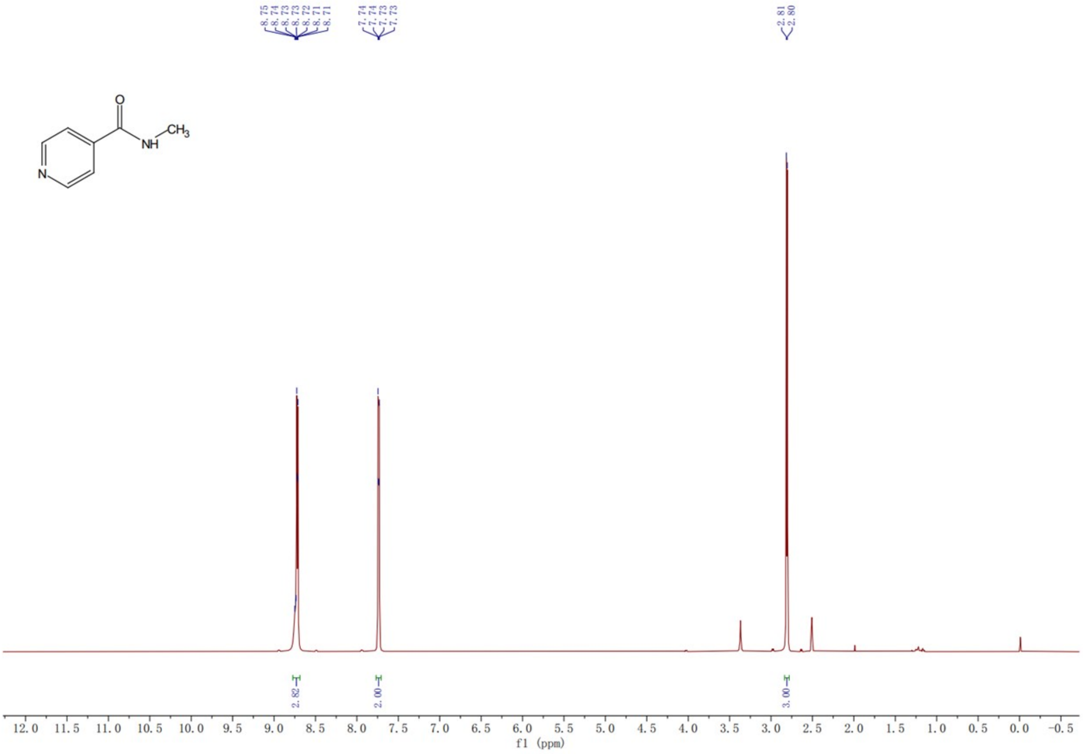

8.75
8.74
8.73
8.73
8.72
8.71
8.71
7.74
7.74
7.73
7.73
2.81
2.80
2.82
2.00
3.00
f1 (ppm)

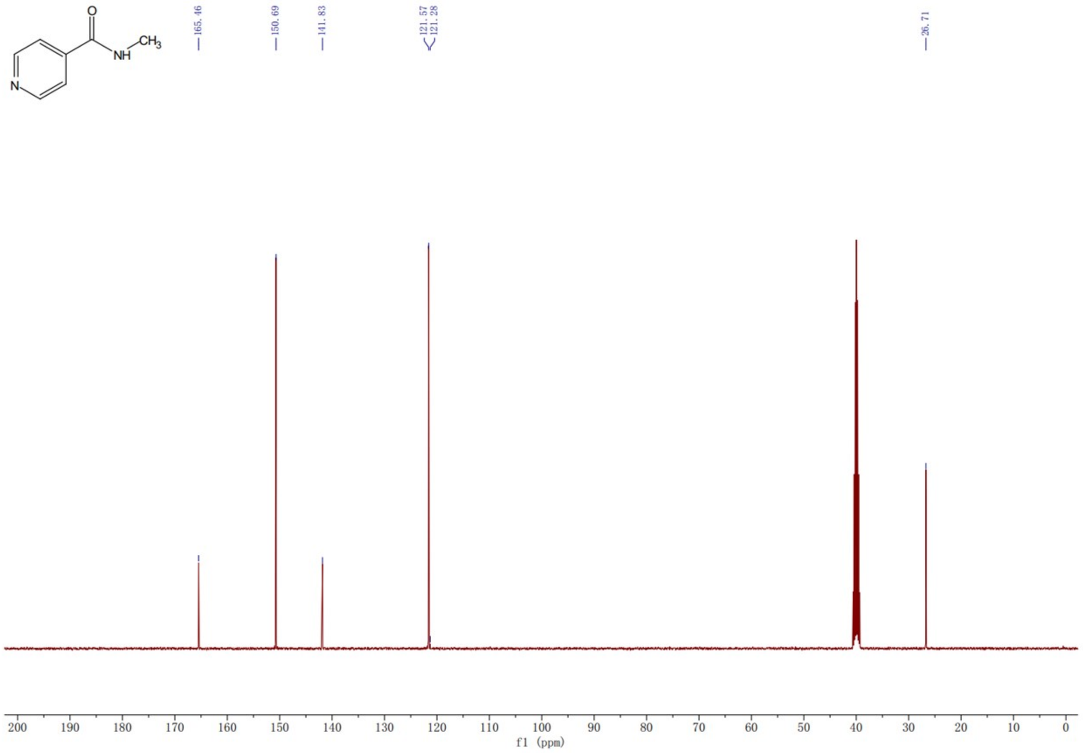

165.46
150.69
141.83
121.57
121.28
26.71
f1 (ppm)

3r

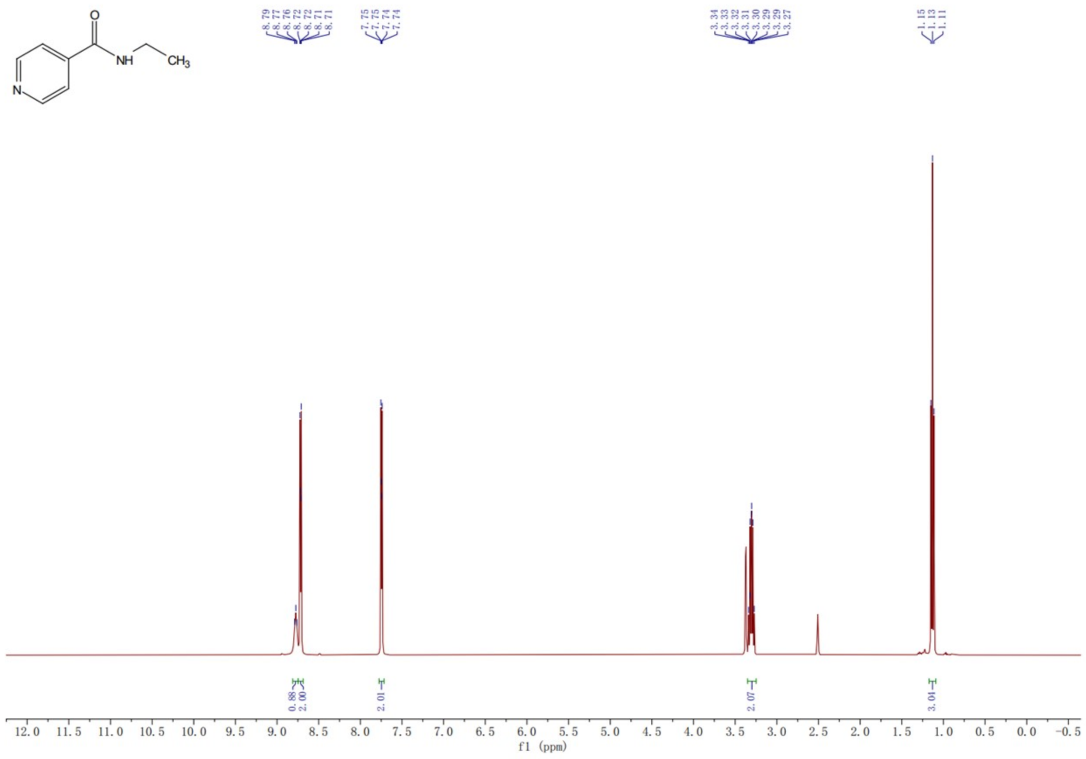

O
NH
CH3
N
8.79 8.77 8.76 8.72 8.72 8.71 8.71
7.75 7.75 7.74 7.74
3.34 3.33 3.32 3.31 3.30 3.29 3.29 3.27
1.15 1.13 1.11
0.88 2.00 2.01 2.07 3.01
12.0 11.5 11.0 10.5 10.0 9.5 9.0 8.5 8.0 7.5 7.0 6.5 6.0 5.5 5.0 4.5 4.0 3.5 3.0 2.5 2.0 1.5 1.0 0.5 0.0 -0.5
f1 (ppm)

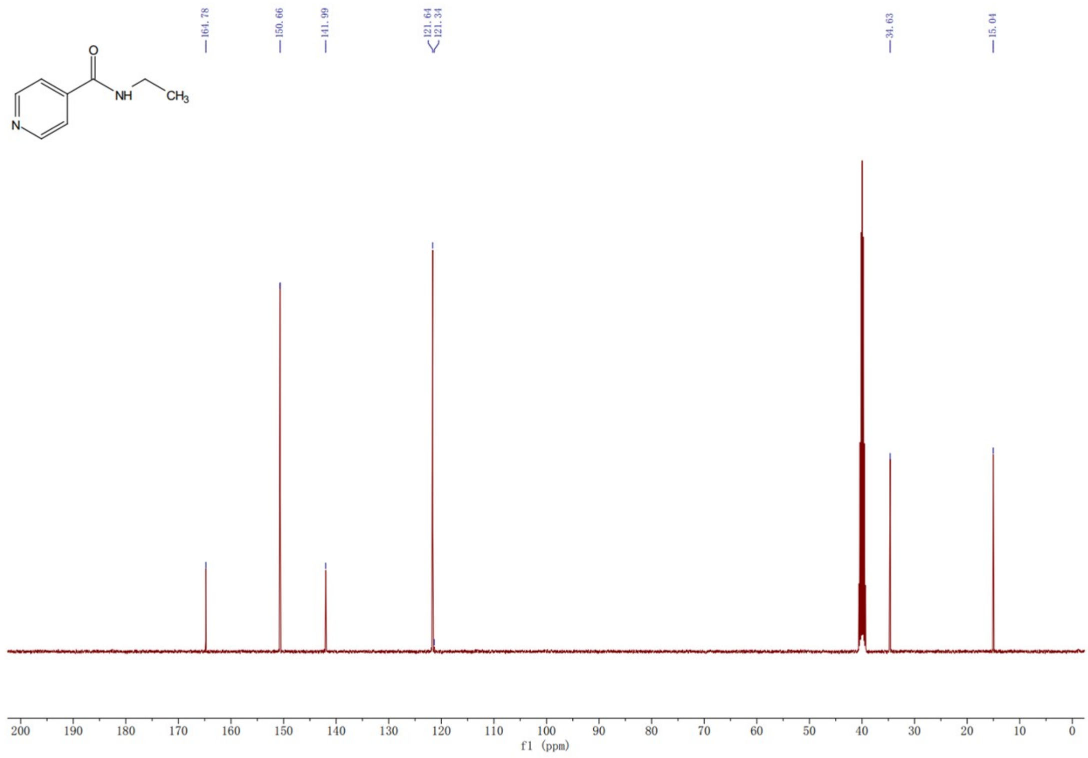

O
NH
CH3
N
164.78
150.66
141.99
121.64 121.34
34.63
15.04
200 190 180 170 160 150 140 130 120 110 100 90 80 70 60 50 40 30 20 10 0
f1 (ppm)

Notes and references

1. M. M. Khan, Saigal, S. Khan, S. Shareef and S. C. Sahoo, *Rsc Advances*, 2018, **8**, 41892-41903.
2. S. Singh, S. Mondal, N. Vodnala and C. K. Hazra, *Green Chemistry*, 2023, **25**, 1014-1022.
3. B. Dam, A. K. Sahoo and B. K. Patel, *Green Chemistry*, 2022, **24**, 7122-7130.
